# Leukocyte Count and Coronary Artery Disease Events in People With Human Immunodeficiency Virus: A Longitudinal Study

**DOI:** 10.1093/cid/ciad033

**Published:** 2023-01-23

**Authors:** Emma F Avery, Julia N Kleynhans, Bruno Ledergerber, Isabella C Schoepf, Christian W Thorball, Neeltje A Kootstra, Peter Reiss, Lene Ryom, Dominique L Braun, Maria C Thurnheer, Catia Marzolini, Marco Seneghini, Enos Bernasconi, Matthias Cavassini, Hélène Buvelot, Roger D Kouyos, Jacques Fellay, Huldrych F Günthard, Philip E Tarr, for the Swiss HIV Cohort Study, A Anagnostopoulos, M Battegay, E Bernasconi, J Boni, DL Braun, HC Bucher, A Calmy, M Cavassini, A Ciuffi, G Dollenmaier, M Egger, L Elzi, J Fehr, J Fellay, H Furrer, CA Fux, HF Gunthard, D Haerry, B Hasse, HH Hirsch, M Hoffmann, I Hosli, M Huber, CR Kahlert, L Kaiser, O Keiser, T Klimkait, RD Kouyos, H Kovari, B Ledergerber, G Martinetti, Tejada B de Martinez, C Marzolini, KJ Metzner, N Muller, D Nicca, P Paioni, G Pantaleo, M Perreau, A Rauch, C Rudin, AU Scherrer, P Schmid, R Speck, M Stockle, P Tarr, A Trkola, P Vernazza, G Wandeler, R Weber, S Yerly

**Affiliations:** University Department of Medicine and Infectious Diseases Service, Kantonsspital Baselland, University of Basel, Bruderholz, Switzerland; University Department of Medicine and Infectious Diseases Service, Kantonsspital Baselland, University of Basel, Bruderholz, Switzerland; Division of Infectious Diseases and Hospital Epidemiology, University Hospital Zurich, University of Zurich, Zurich, Switzerland; University Department of Medicine and Infectious Diseases Service, Kantonsspital Baselland, University of Basel, Bruderholz, Switzerland; Department of Infectious Diseases, Bern University Hospital, University of Bern, Bern, Switzerland; Hepatology, Department for Visceral Surgery and Medicine, Bern University Hospital, University of Bern, Bern, Switzerland; Precision Medicine Unit, Lausanne University Hospital and University of Lausanne, Lausanne, Switzerland; Department of Experimental Immunology, Amsterdam University Medical Centers, University of Amsterdam, Amsterdam, The Netherlands; Department of Global Health and Division of Infectious Disease, Amsterdam University Medical Centers, University of Amsterdam, and Amsterdam Institute for Global Health and Development, Amsterdam, The Netherlands; Centre of Excellence for Health, Immunity, and Infections, Rigshospitalet, University of Copenhagen, Copenhagen, Denmark; Division of Infectious Diseases and Hospital Epidemiology, University Hospital Zurich, University of Zurich, Zurich, Switzerland; Institute of Medical Virology, University of Zurich, Zurich, Switzerland; Department of Infectious Diseases, Bern University Hospital, University of Bern, Bern, Switzerland; Division of Infectious Diseases and Hospital Epidemiology, University Hospital Basel, Basel, Switzerland; Division of Infectious Diseases, Kantonsspital St Gallen, St. Gallen, Switzerland; Division of Infectious Diseases, Ospedale Regionale Lugano, University of Geneva and Università della Svizzera italiana, Lugano, Switzerland; Infectious Diseases Service, Lausanne University Hospital, University of Lausanne, Lausanne, Switzerland; Division of Infectious Disease, Geneva University Hospital, Geneva, Switzerland; Division of Infectious Diseases and Hospital Epidemiology, University Hospital Zurich, University of Zurich, Zurich, Switzerland; Institute of Medical Virology, University of Zurich, Zurich, Switzerland; Precision Medicine Unit, Lausanne University Hospital and University of Lausanne, Lausanne, Switzerland; School of Life Sciences, Ecole Polytechnique Fédérale de Lausanne, Lausanne, Switzerland; Division of Infectious Diseases and Hospital Epidemiology, University Hospital Zurich, University of Zurich, Zurich, Switzerland; Institute of Medical Virology, University of Zurich, Zurich, Switzerland; University Department of Medicine and Infectious Diseases Service, Kantonsspital Baselland, University of Basel, Bruderholz, Switzerland

**Keywords:** HIV infection, coronary artery disease, leukocytes, multivariable analysis, white blood cells

## Abstract

**Background:**

People with human immunodeficiency virus (HIV; PWH) have increased cardiovascular risk. Higher leukocyte count has been associated with coronary artery disease (CAD) events in the general population. It is unknown whether the leukocyte-CAD association also applies to PWH.

**Methods:**

In a case-control study nested within the Swiss HIV Cohort Study, we obtained uni- and multivariable odds ratios (OR) for CAD events, based on traditional and HIV-related CAD risk factors, leukocyte count, and confounders previously associated with leukocyte count.

**Results:**

We included 536 cases with a first CAD event (2000–2021; median age, 56 years; 87% male; 84% with suppressed HIV RNA) and 1464 event-free controls. Cases had higher latest leukocyte count before CAD event than controls (median [interquartile range], 6495 [5300–7995] vs 5900 [4910–7200]; *P* < .01), but leukocytosis (>11 000/µL) was uncommon (4.3% vs 2.1%; *P* = .01). In the highest versus lowest leukocyte quintile at latest time point before CAD event, participants had univariable CAD-OR = 2.27 (95% confidence interval, 1.63–3.15) and multivariable adjusted CAD-OR = 1.59 (1.09–2.30). For comparison, univariable CAD-OR for dyslipidemia, diabetes, and recent abacavir exposure were 1.58 (1.29–1.93), 2.19 (1.59–3.03), and 1.73 (1.37–2.17), respectively. Smoking and, to a lesser degree, alcohol and ethnicity attenuated the leukocyte-CAD association. Leukocytes measured up to 8 years before the event were significantly associated with CAD events.

**Conclusions:**

PWH in Switzerland with higher leukocyte counts have an independently increased risk of CAD events, to a degree similar to traditional and HIV-related risk factors.

## Teaser

Leukocyte count is associated with coronary artery disease (CAD) events in the general population. Here we show that leukocytes are independently associated with CAD events in people with HIV in Switzerland, after adjusting for traditional and HIVrelated risk factors.

People with human immunodeficiency virus (HIV; PWH), have an increased risk for coronary artery disease (CAD) events compared with the general population [1, 2]. CAD risk in PWH is related to traditional CAD risk factors, HIV-related factors including chronic inflammation [3, 4], immunosuppression [5, 6], potential deleterious effects of certain antiretroviral therapy (ART) agents [7, 8], and individual genetic background [9]. An increased CAD risk may persist in PWH with suppressed HIV viremia [1, 2]. This suggests a role for low-level inflammation and immune activation in the pathogenesis of CAD in PWH and has generated considerable interest in inflammatory biomarkers for CAD event prediction in PWH [4, 10, 11].

Leukocytes are implicated in the pathogenesis of atherosclerosis, and ever since the 1980s, studies in the general population have shown leukocyte count in the peripheral blood to be an independent risk factor for CAD events [12–15]. Whether blood leukocytes are associated with CAD events in PWH has not been verified. Therefore, the aim of this report is to assess an independent association of leukocyte count with CAD events in participants of the Swiss HIV Cohort Study (SHCS), analyzed in the context of traditional and HIV-related CAD risk factors. We also considered multiple factors that may influence leukocytes, including ethnicity [16], smoking [17], infections, and alcohol intake [18].

## METHODS

### Study Population

We included PWH enrolled in the SHCS (http://www.shcs.ch [19]), an observational study that has prospectively enrolled PWH since 1988, and has captured rich cardiovascular, metabolic, genetic, and other data since 1999. Participants provided written informed consent. The study was approved by the local ethics committees. Cases had a first CAD event and controls were CAD event-free during the study period (1 January 2000–31 October 2021).

### CAD Events

CAD events were defined per the Data Collection on Adverse events of Anti-HIV Drugs study and the World Health Organization's Monitoring Trends and Determinants in Cardiovascular Disease Project [20], as we have previously published [9, 21]. CAD events included myocardial infarction, coronary angioplasty/stenting, coronary artery bypass grafting, and fatal cases (confirmed at autopsy or ascertained by the treating HIV physician as sudden death with no other likely cause plus evidence of CAD before death).

### Case-Control Matching

As in our previous CAD case-control studies [9, 21], we used incidence density sampling [22], aiming to select 1 to 3 event-free controls for each case. We used risk-set sampling [23 (ie, we matched controls at the CAD event date of the corresponding cases [matching date] on similar observation duration, and their observation period was during similar calendar periods to account for differences in ART [with different CAD risk associations] [8, 24]) in use during different periods and other differences. Matching criteria were sex, age ± 4 years, and date of SHCS registration ± 4 years. Observation time started at SHCS registration; observation ended for cases at the matching date (CAD event date) and for controls ended at the first regular SHCS follow-up visit after the matching date, respectively.

### Power Calculation

To capture odds ratios of ≥1.6, we would need 255 cases and 2 controls per case [30], assuming an exposure correlation between pairs in the case-control set of 0.2 [30].

### Leukocyte Count

The SHCS database routinely includes total leukocytes, total lymphocytes, CD4, and CD8 counts. For the main analysis, we compared latest leukocyte count before the matching date in cases and controls and. In addition, we considered leukocyte count at increasing intervals before the matching date. In exploratory analyses, we obtained neutrophils, eosinophils, and the neutrophil-lymphocyte ratio retrospectively for participants at University Hospital Zurich, where approximately 20% of SHCS participants are followed.

### Clinical CAD Risk Factors

Covariables were defined a priori based on their CAD association in the general population, as reported previously [9, 21, 25], and were ascertained at the latest SHCS visit before the matching date except for CD4 nadir (lowest CD4 value during the study period). Covariables included age (per 10 years older, added to detect any residual effect of suboptimal matching, as we have done previously [21]), family history of CAD, smoking, diabetes mellitus, hypertension, and dyslipidemia (total cholesterol >6.2 mmol/L or high-density lipoprotein < 1 mmol/L [men] and <1.2 mmol/L [women] or use of lipid-lowering drugs [25]). HIV-related covariables included HIV RNA < or ≥50 copies/mL, CD4 nadir, and ART exposures until the matching date, based on their CAD association in the Data Collection on Adverse events of Anti-HIV Drugs study [8, 24], including recent (past 6 months) abacavir, didanosine, and integrase inhibitors; and cumulative (>1 year) exposure to lopinavir, indinavir, boosted darunavir, and stavudine [9]; hepatitis C [26]; and cytomegalovirus seropositivity [27].

### Potential Confounding Variables Associated With Leukocyte Count

These were defined a priori, based on reported associations in the general population. We considered both current smoking (vs past/never [28]) and daily cigarettes smoked (never, not currently, ≤5/d, 6–20/d, >20/d, unknown [29]); ethnicity (White/Black/Hispanic/Asian) [16]; and alcohol (none/mild vs moderate/heavy; defined in the SHCS until 2012 as </≥40 g [men], </≥20 g [women]), and using the Alcohol Use Disorders Identification Test-C questionnaire beginning in 2013 (</≥4 points [men], </≥2 points [women]; hepatitis.va.gov/alcohol/treatment/audit-c.asp#S1X) [18]. We also tested each variable in the CAD event model for a potential interaction with leukocytes (Supplementary Methods, Supplementary Table 1). We did not analyze corticosteroid use and non-HIV inflammatory conditions because these were recorded before the event in only 8 cases/36 controls and in 3 cases/5 controls, respectively, and because of insufficient available details (eg, specific diagnoses, date, corticosteroid duration/dose).

### Infection Episodes

Because infections may influence leukocytes, we assessed nonopportunistic infections (recorded in the SHCS since 2017, defined as leading to hospitalization or antibiotic use for ≥5 days) and opportunistic infections in the year before matching date in cases and controls.

### Sensitivity Analyses

To test the robustness of the leukocyte-CAD association; (1) we replaced all risk factors by the 10-year Framingham risk score (FRS) for CAD or (2) by FRS risk category (<10% vs ≥ 10% risk); (3) analysis restricted to participants with suppressed HIV RNA at matching date; and (4) after adding the latest estimated glomerular filtration rate (eGFR) before CAD event to the model (note that kidney function is available in the SHCS after 1 January 2002).

### Statistical Analyses

Characteristics of cases and controls were compared using Fisher exact test (categorical variables) and Wilcoxon rank-sum test (continuous variables). Univariable, bivariable, and multivariable conditional logistic regression analyses were used to estimate associations of the different risk factors with CAD and their interactions. We decided a priori to stratify leukocyte counts into quintiles for better visualization of potentially nonlinear associations with CAD events. Variables were entered into the multivariable model if their association in the univariable model had a *P* level < .2. Model fit and interactions were analyzed using Akaike and Bayesian information criteria and likelihood ratio tests. The effect of potential confounders on the leukocyte-CAD association was tested on a 1:1 basis (bivariable models including interaction terms). Trajectories of total leukocytes, leukocyte subtypes, and smoking over the past 15 years were created using local polynomial smoothing with the Epanechnikov kernel. We used Stata/SE 17.0 (StataCorp, College Station, TX, USA).

## RESULTS

### Participants: CAD Events

**Figure 1.**
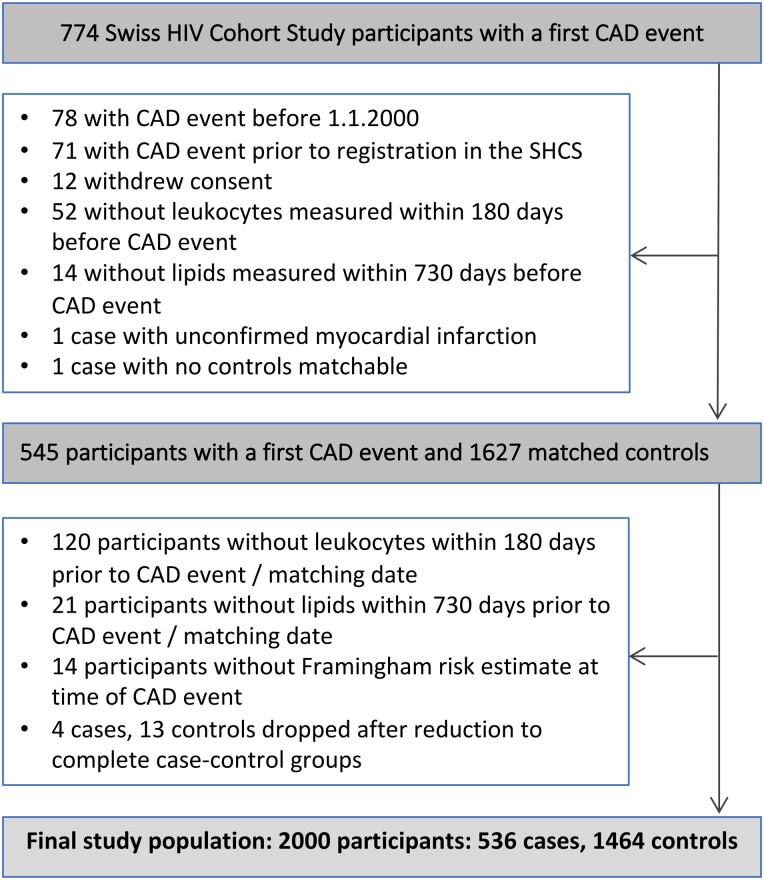
Study flowchart. Abbreviations: CAD, coronary artery disease; MI, myocardial infarction.

Participant disposition is shown in Figure 1 and participants' characteristics in Table 1. The final study population included 2000 participants (ie, 536 cases with a first CAD event and 1464 matched CAD event-free controls). Registration of participants started in January 1985, and CAD events were considered until August 2021. The median (interquartile range [IQR]) date of CAD events was 3 May 2013 (5 October 2007–18 September 2017), and the median (IQR) duration of observation was 13.1 (8.1–19.2) years. CAD events included myocardial infarction (n = 274), coronary angioplasty/stenting (n = 211), coronary artery bypass grafting (n = 39), and fatal CAD cases (n = 12) [21]. Cases were more likely to be smokers, diabetic, dyslipidemic, hypertensive, or have a CAD family history (Table 1).

**Table 1.** Characteristics of Cases and Controls

		All Participants (N = 2000)	Cases (n = 536)	Controls (n = 1464)	*P* Values
Male, n (%)^a^		1734 (86.7)	464 (86.6)	1270 (86.8)	.94^c^
Age at CAD event (y), median (IQR)^a^		56 (49–63)	56 (49–63)	56 (49–62)	.54^d^
Date of CAD event, median (IQR)^a^		3 May 2013 (5 October 2007–18 September 2017)	21 April 2013 (10 September 2007–15 September 2017)	15 May 2013 (30 October 2007–18 September 2017)	.83^d^
Duration of observation (y), median (IQR)^a^		13.1 (8.1–19.2)	13.2 (8.2–19.2)	13.1 (8.0–19.2)	.97^d^
Ethnicity, n (%)	White	1876 (93.8)	514 (95.9)	1362 (93.0)	.07^c^
	Black	78 (3.9)	14 (2.6)	64 (4.4)	…
	Hispanic	19 (1.0)	5 (0.9)	14 (0.9)	…
	Asian	27 (1.4)	3 (0.5)	24 (1.6)	…
HIV acquisition mode, *n* (%)	Heterosexual	589 (29.5)	162 (30.2)	427 (29.2)	.14^c^
	MSM	1008 (50.4)	258 (48.1)	750 (51.2)	…
	IDU	335 (16.8)	103 (19.2)	232 (15.9)	…
	Other	68 (3.4)	13 (2.4)	55 (3.8)	…
Smoking status, n (%)	Current	847 (42.4)	285 (53.2)	562 (38.4)	<.01^c^
	Past	599 (30)	146 (27.2)	453 (30.9)	…
	Never	554 (27.7)	105 (19.6)	449 (30.7)	…
Cigarettes smoked per day, number of smokers, n (%)	≤5 cig/d	113 (5.7)	28 (5.2)	85 (5.8)	<.01^c^
	6–20 cig/d	527 (26.4)	194 (36.2)	333 (22.8)	…
	>20 cig/d	183 (9.2)	55 (10.3)	128 (8.7)	…
	Unknown	24 (1.2)	8 (1.5)	16 (1.1)	…
Cocaine use intravenously and not intravenously, n (%)	Recent^b^	77 (3.9)	23 (4.3)	54 (3.7)	.86^c^
	Ever	(8.5)	45 (8.4)	124 (8.5)	…
Alcohol use, last recorded before endpoint, n (%)	None/mild	1492 (88)	404 (89.6)	1088 (87.5)	.27^c^
	Moderate/heavy	203 (12)	47 (10.4)	156 (12.5)	…
Education level, n (%)	Mandatory school	331 (16.6)	96 (17.9)	235 (16.1)	.26^c^
	Apprenticeship	947 (47.4)	264 (49.3)	683 (46.7)	…
	Higher education	619 (47.4)	154 (28.7)	465 (31.8)	…
	Other/ missing	103 (5.2)	22 (4.1)	81 (5.5)	…
Family history of CAD, n (%)		226 (11.3)	87 (16.2)	139 (9.5)	<.01^c^
Diabetes mellitus, n (%)		199 (10)	80 (14.9)	119 (8.1)	<.01^c^
Hypertension, n (%)		613 (30.7)	190 (35.5)	423 (28.9)	.01^c^
Dyslipidemia, n (%)		1026 (51.3)	319 (59.5)	707 (48.3)	<.01^c^
CMV seropositivity, n (%)		1712 (85.6)	1239 (84.6)	473 (88.3)	.04^c^
Hepatitis C seropositivity, n (%)		457 (22.9)	135 (25.2)	322 (22.0)	.13^c^
Framingham risk score (10-y risk), n (%)	<10%	803 (40.2)	142 (26.5)	661 (45.2)	<.01^c^
	10%–20%	815 (40.8)	240 (44.8)	575 (39.3)	…
	>20%	382 (19.1)	154 (28.7)	228 (15.6)	…
Leukocytes/µL, median (IQR)	Latest before CAD event	6020 (5000–7460)	6495 (5300–7995)	5900 (4910–7200)	<.01^c^
	1 y before CAD event	5900 (4900–7200)	6200 (5040–7700)	5800 (4810–7100)	<.01^d^
	2 y before CAD event	5900 (4820–7300)	6145 (5000–7600)	5800 (4800–7200)	<.01^d^
	3 y before CAD event	5800 (4770–7100)	5920 (4900–7400)	5780 (4700–7000)	.01^d^
	5 y before CAD event	5755 (4600–7080)	5880 (4685–7605)	5700 (4525–6900)	.04^d^
	8 y before CAD event	5600 (4500–6860)	5800 (4590–7400)	5500 (4500–6690)	.06^d^
	9 y before CAD event	5500 (4500–6990)	5600 (4410–7100)	5500 (4535–6920)	.69^d^
	10 y before CAD event	5495 (4500–7000)	5500 (4400–7000)	5450 (4500–6910)	.72^d^
CD4 at matching date, median (IQR)		545 (389–762)	546 (384–769)	545 (390–760)	.86^d^
CD4 nadir (cells/μL), median (IQR)		166 (72–261)	158 (64–254)	170 (78–265)	.8^d^
CD4 nadir <50 cells/μL, n (%)		356 (17.8)	107 (20.0)	249 (17.0)	.13^c^
Previous AIDS, n (%)		566 (28.3)	161 (30.0)	405 (27.7)	.31^c^
On ART, n (%)		1893 (94.7)	519 (96.8)	1374 (93.9)	<.01^c^
On ART, HIV RNA <50 copies/mL (undetectable), n (%)		1702 (85.1)	452 (84.3)	1250 (85.4)	<.01^c^
Total years on ART before CAD event, median (IQR)		11.7 (6.5–17.7)	12 (7.5–18.5)	11.5 (6.2–17.5)	<.01^d^
Received Abacavir in 6 m before CAD event, n (%)		500 (25)	173 (32.3)	327 (22.3)	<.01^c^
Received didanosine in 6 m before CAD event, n (%)		66 (3.3)	26 (4.9)	40 (2.7)	.02^c^
Received an integrase inhibitor in 6 m before CAD event, n (%)		477 (23.9)	142 (26.5)	335 (22.9)	.10^c^
Lopinavir, exposure ≥1 y, n (%)		514 (25.7)	151 (28.2)	363 (24.8)	.13^c^
Indinavir, exposure ≥1 y, n (%)		402 (20.1)	114 (21.3)	288 (19.7)	.45^c^
Darunavir, exposure ≥1 y, n (%)		320 (16.0)	91 (17.0)	229 (15.6)	.49^c^
Stavudine, exposure ≥1 y, n (%)		727 (36.4)	225 (42.0)	502 (34.3)	<.01^c^

All data shown apply to the matching date and are number (%) of participants, unless otherwise indicated.

Abbreviations: AIDS, acquired immunodeficiency virus; ART, antiretroviral therapy; CAD, coronary artery disease; cig, cigarette; CMV, cytomegalovirus; HIV, human immunodeficiency virus; IDU, intravenous drug use; IQR, interquartile range; MSM, men who have sex with men.

Age, sex, date of CAD event, and observation duration were matching criteria. Because of residual imbalance, median age of cases was 0.27 years older than controls (*P* < .01).

In 6 mo before matching date.

Fisher exact test.

Wilcoxon rank-sum test.

### Latest Leukocyte Count: Observed Data

**Figure 2.**
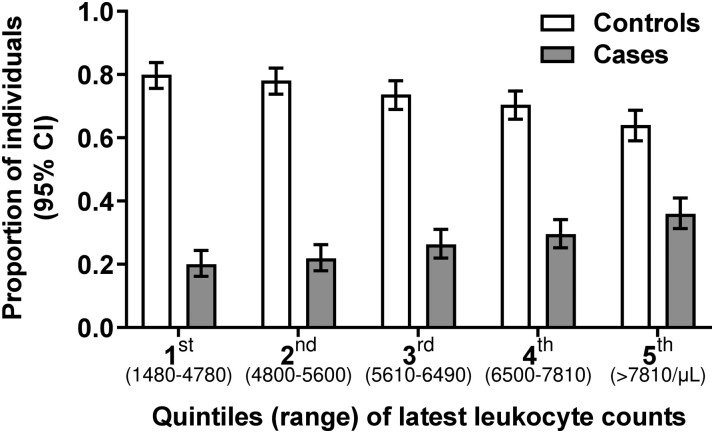
Distribution of leukocyte count in 1464 controls without coronary artery events (white bars) and in 536 cases with coronary artery events (gray bars). We divided CAD cases and CAD event-free controls into 5 quintiles according to their clinical CAD risk and their latest leukocyte count before the matching date. We show here the number, percentage, and 95% confidence intervals of participants in each quintile, plus the range of leukocyte counts in each quintile. Distribution of cases and controls according to latest leukocyte count before matching date. There were 78 (20.1%) cases versus 311 (79.9%) controls in the first quintile, 90 (21.9%) versus 321 (78.1%) in the second quintile, 100 (26.3%) versus 280 (73.7%) in the third quintile, 124 (29.5%) versus 296 (70.5%) in the fourth quintile, and 144 (36%) versus 256 (64%) in the fifth quintile. Abbreviations: CAD, coronary artery disease; CI, confidence interval.

Median time from the latest leukocyte measurement to CAD event (matching date) was 56 (IQR, 30–94) days in cases and 60 (IQR, 29–91) days in controls. Latest median leukocyte count before the matching date was higher in cases than controls (*P* < .01; Table 1). Leukocytosis (>11 000/µL) was uncommon but more frequent in cases than controls (4.3% vs 2.1%; *P* = .01). Figure 2 shows the range of leukocytes in each leukocyte quintile and how the number of cases increases and the number of controls decreases in the higher leukocyte quintiles. Supplementary Table 2 shows leukocytes for cases and controls in each quintile.

### Longitudinal Leukocyte Values: Observed Data

**Figure 3.**
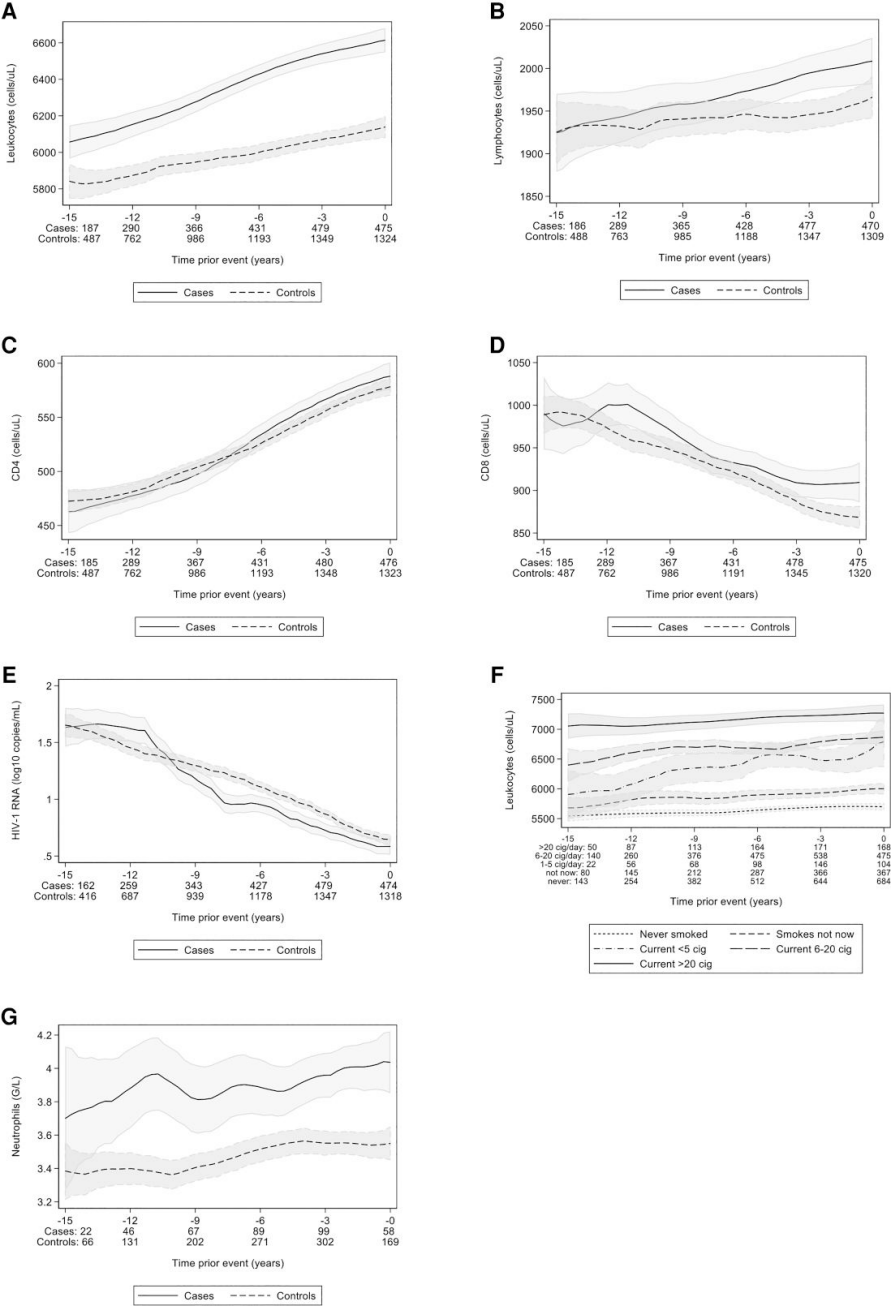
*A*–*G*, Descriptive longitudinal trends for leukocyte count, leukocyte subsets, HIV-RNA, and neutrophil count in cases and controls. Descriptive (observed) trajectories of total leukocytes and different leukocyte subtypes (*A*–*D*) and HIV RNA (*E*) over time for controls versus cases. The lines show the cell counts and the shaded areas denote the 95% confidence intervals created with local polynomial smoothing. We considered only parameters that were from regular (per protocol) 6-monthly follow-up SHCS visits up until 1 d before the CAD event (cases) and matching date (controls). *F*, The leukocyte count stratified by different smoking amount categories. *G*, The observed trajectories of total neutrophils for the University of Zurich subpopulation over time for controls versus cases. The graphs portray an open cohort design (all participants are included, regardless of observation duration). The graphs portraying a closed cohort (in which only participants with ≥15-y observation time are included) can be found in the Supplementary Figure 1. Abbreviations: CAD, coronary artery disease; cig, cigarettes.

Median (IQR) leukocyte count was higher in cases than controls at 1 year (*P* < .01), 2 years (*P* < .01), 3 years (*P* < .01), and 5 years (*P* = .04) before a CAD event, but not at 8, 9, and 10 years before a CAD event (*P* = .06, *P* = .69, and *P* = .72, respectively). Longitudinal observed leukocyte trajectories are shown in Figure 3*A*. Longitudinal trajectories of observed total lymphocytes (Figure 3*B*), CD4 cells (Figure 3*C*), CD8 cells (Figure 3*D*), and observed HIV-RNA trajectories (Figure 3*E*) were similar in cases and controls. Longitudinal leukocyte trajectories in smokers versus nonsmokers showed an apparent dose relation regarding cigarettes smoked per day (Figure 3*F*).

### Leukocyte Count and CAD Events: Univariable Model

**Figure 4.**
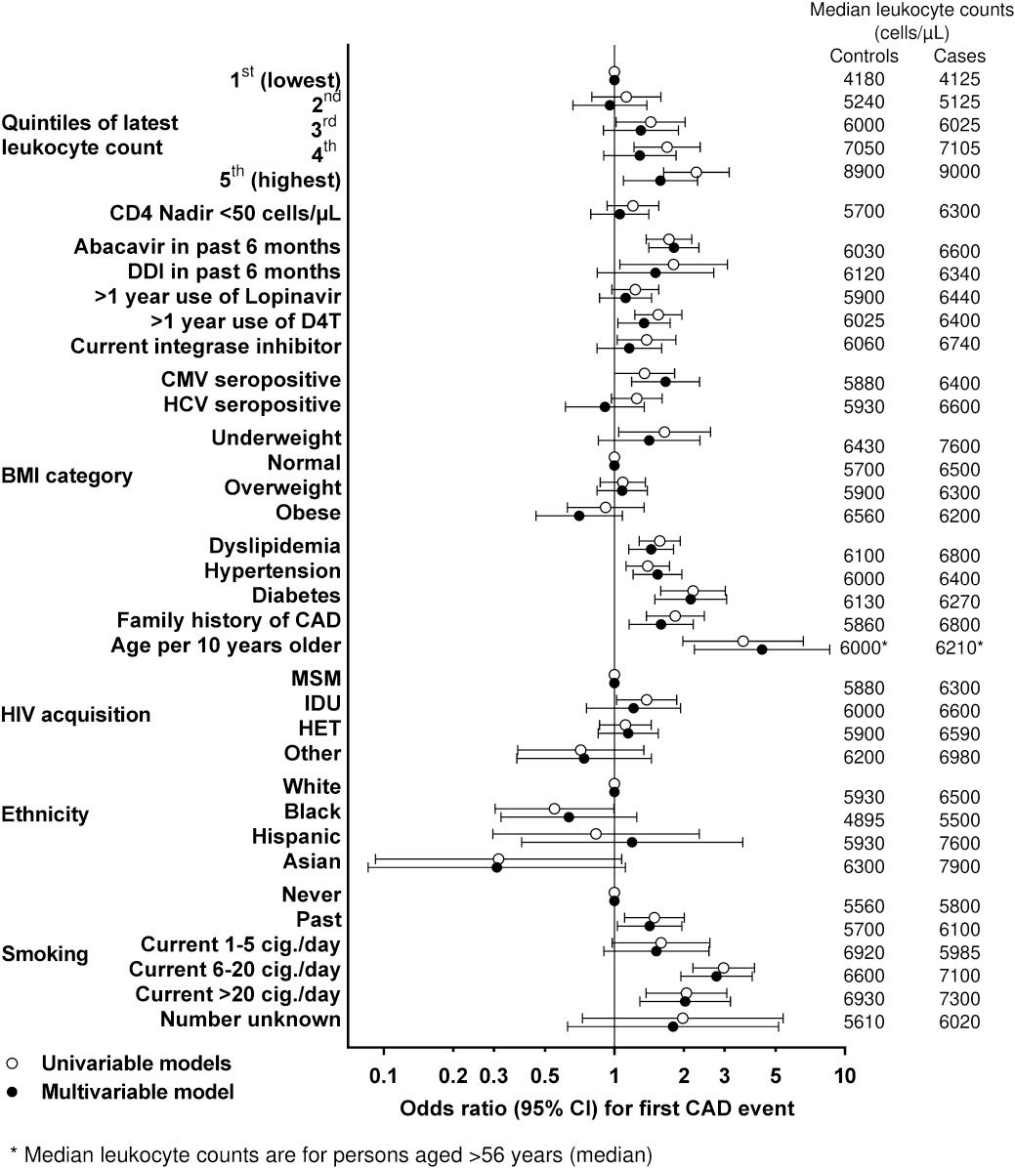
ORs for CAD events (with 95% CIs), according to individual clinical risk factors and latest leukocyte quintiles. Results show univariable and bivariable conditional logistic regression of associations of latest leukocyte count with CAD events for 536 cases and 1464 controls. Latest leukocyte count (fifth [highest] vs first [lowest] was significantly associated with CAD events in univariable analysis and in multivariable analysis [ie, adjusted for all variables shown]). Note: All odds ratios and 95% CIs shown in Figure 4 are also tabulated in Supplementary Table 3. The right-hand panel shows median leukocyte counts (cells/µL) in cases and controls in the different categories. Abbreviations: CAD, coronary artery disease; CI, confidence interval; OR, odds ratio.

In the latest sample before a CAD event, leukocyte count was associated with CAD events (per 1000 leukocytes higher, CAD-OR = 1.11; 95% confidence interval [CI], 1.05–1.16). Compared with participants in the first (lowest) leukocyte quintile, participants in the second, third, fourth, and fifth (highest) quintile had univariable CAD-OR = 1.13 (95% CI, .80–1.59), 1.44 (1.02–2.03), 1.69 (1.22–2.35), and 2.27 (1.64–3.15), respectively. For comparison, univariable CAD-OR for hypertension, dyslipidemia, diabetes, and recent abacavir exposure was 1.40 (1.12–1.73), 1.58 (1.29–1.93), 2.19 (1.59–3.03), and 1.73 (1.37–2.17), respectively. Univariable associations of all individual risk factors with CAD are shown in Figure 4 and Supplementary Table 3.

### Longitudinal Leukocyte Counts and CAD Events: Univariable Model

Leukocyte count (fifth vs first quintile) remained significantly associated with CAD events when measured at year −1 (CAD-OR = 1.81; 95% CI, 1.30–2.53; n = 1896 participants), year −2 (1.66; 1.18–2.33; *n* = 1749), year −3 (1.56; 1.09–2.22; n = 1617), year −5 (1.74; 1.14–2.64; n = 1231), year −8 (2.18; 1.24–3.84; n = 657), but not at year −9 (1.12; 0.59–2.13; n = 499), or year −10 (0.66; 0.33–1.35; n = 369) before the CAD event (Supplementary Table 4).

### Leukocyte Count and CAD Events: Multivariable Model

In the final model, participants had increased adjusted CAD risk in the fifth (highest) leukocyte quintile (ie, participants in the second, third, fourth, and fifth vs first [lowest] quintile had adjusted CAD-OR = 0.96 [95% CI, .66–1.38], 1.30 [0.90–1.90], 1.29 [0.90–1.85], and 1.59 [1.09–2.30], respectively) (Figure 4, Supplementary Table 3). For comparison, multivariable CAD-OR for hypertension, dyslipidemia, diabetes, and recent abacavir exposure were 1.54 (1.21–1.97), 1.44 (1.15–1.81), 2.15 (1.5–3.07), and 1.81 (1.41–2.33), respectively.

### Leukocyte Count and CAD Events: Potential Confounders

Median leukocyte count was higher in cases than controls in most confounder categories (Figure 4). In individual 1:1 bivariable analyses (Table 2, Supplementary Table 1), last leukocyte count remained associated with CAD events but the association was attenuated when we added smoking status (fifth vs first leukocyte quintile, CAD-OR = 1.85 [1.31–2.61]) or the latest number of cigarettes smoked per day (fifth vs first leukocyte quintile, CAD-OR = 1.82 (1.30–2.56); *P* < .01), suggesting that smoking in part explains the leukocyte-CAD association. The leukocyte-CAD event association was attenuated to a lesser degree by alcohol or when considering ethnicity. Attenuation was minimal for the other individual confounders. All interactions were discarded because of lack of significance (all *P* > .05; Table 2, Supplementary Table 1).

**Table 2.** Bivariable Analyses Showing CAD Odds Ratio (95% CI) for Fifth (Highest) Versus First (Lowest) Leukocyte Quintile, With 1:1 Addition of Individual Variables That May Influence Leukocyte Count

Variable	CAD Odds Ratio (95% CI) for Fifth (Highest) vs First (Lowest) Leukocyte Quintile	Likelihood Ratio Test for Interaction
Univariable analysis		
Leukocytes, fifth (highest) vs first (lowest) quintile	2.27 (1.63–3.15)	–
Individual bivariable analyses (leukocyte quintiles plus individual variables added 1:1)		
+ Smoking status (current vs previous vs never)	1.82 (1.30–2.56)	0.130
+ Number of cigarettes smoked daily	1.85 (1.31–2.61)	0.589
+ Ethnicity	2.21 (1.59–3.07)	0.861
+ Last alcohol intake: moderate/heavy	1.98 (1.39–2.81)	0.351

All variables were associated with the CAD-odds ratio and had *P* < .01.

Abbreviations: CAD, coronary artery disease; CI, confidence interval.

### Leukocyte Count and Infection Episodes

There were 12 cases and 26 controls with an opportunistic infection (OI) in the year before CAD event (*P* = .47; Supplementary Table 5). Serious non-OIs were documented in 15/163 (9.2%) cases versus 21/447 (4.7%) controls (*P* = .05). Participants with/without OI (*P* = .64) and with/without serious non-OI (*P* = .59) had similar median latest leukocytes (Supplementary Table 5).

### Sensitivity Analyses With FRS

After adjustment for FRS, leukocyte count remained associated with CAD events. Participants in the fifth versus first leukocyte quintile had CAD-OR = 1.64 (1.16–2.32) when adjusting for FRS, and CAD-OR = 1.82 (1.30–2.56) when adjusting for FRS category (≥10% vs <10%; Supplementary Table 6*A* and *B*).

### Sensitivity Analysis: Participants With Suppressed HIV RNA

In multivariable analysis restricted to participants with suppressed viremia at the latest pre-event time point (n = 1559 participants), results remained essentially unchanged; participants in the fifth versus first leukocyte quintile had CAD-OR = 1.63 (1.06–2.50) (Supplementary Table 7).

### Leukocyte Subsets and CAD Events: Univariable Models

Leukocyte subsets were available for the 517/2000 participants followed at University Hospital Zurich. Latest median neutrophil count was higher in 132 cases versus 385 controls (*P* < .01) (ie, 3835/µL [IQR, 2800–4925] vs 3220/µL [IQR, 2470–4230]). Longitudinal observed neutrophil count showed divergent trajectories in cases and controls up to 12 years before a CAD event (Figure 3*G*). Zurich participants in the fifth versus first leukocyte quintile had CAD-OR = 4.78 (2.31–9.87), and in the fifth versus first neutrophil quintile had CAD-OR = 2.19 (1.13–4.26; Supplementary Table 8). Because of the high correlation between leukocytes and neutrophils (Spearman rho = 0.85, *P* < .01), results from simultaneous modeling (leukocytes and neutrophils in the same model) cannot be interpreted. We found no evidence of an association of eosinophil count or neutrophil:lymphocyte ratio with CAD events (data not shown).

### Sensitivity Analysis Including eGFR

eGFR (available pre-CAD event in 1546/2000 participants) was associated with CAD events (univariable CAD-OR = 1.15 [1.08–1.23] per 10 mL/min/1.73 m^2^ lower eGFR; Supplementary Table 9). When we included latest eGFR in the final model, participants in the fifth versus first leukocyte quintile had CAD-OR = 1.52 (1.00–2.31) (ie, CAD-OR was essentially unchanged but the 95% CI was wider).

## DISCUSSION

Multiple studies have recorded associations of CAD with biomarkers of inflammation and coagulation in PWH [4, 10, 11] and multiple studies document associations of CAD with leukocyte count in the general population [12–15]. To our knowledge, this is the first report of an independent association of leukocyte count with CAD events in PWH. Our study has 3 main findings: first, participants with the highest leukocytes (top quintile, >7810/µL) had a 1.59-fold increased CAD event risk in the final multivariable model. This effect size of high leukocytes was similar to the effect of established CAD risk factors, including hypertension, diabetes, dyslipidemia, or recent abacavir exposure. Second, as in the general population, leukocyte count within normal range values was a predictor of CAD events and overt leukocytosis was infrequent. Third, the leukocyte-CAD association was in part explained by smoking, a well-recorded CAD risk factor known to increase leukocytes [17]. Although the association of black ethnicity or alcohol with lower leukocytes is well established [16, 18], these factors only minimally modified the leukocyte-CAD association in our study. The contribution of leukocyte count to CAD events in PWH may demonstrate the potential clinical value of monitoring leukocytes, a cheap, routinely available biomarker with short turnaround time. Although this was beyond the scope of our study (this will require prospective trials), our findings suggest how knowledge of chronically elevated leukocytes increasing CAD event risk by >50% in the 20% PWH in the top leukocyte quintile may motivate clinicians to place even more emphasis on the optimization of cardiovascular risk factors, and, perhaps, primary prevention of CAD with statins in such persons.

Our result of an independent association of leukocyte count with CAD events in PWH appears robust because it persisted after consideration of traditional and HIV-associated CAD risk factors, and in sensitivity analyses adjusting for FRS. Additional strengths of our study are the inclusion of only leukocyte values taken until the day before the CAD event to address the issue of reverse causation (ie, leukocytes being elevated because of a CAD event). In addition, we included all CAD events that occurred in the well-established SHCS over a >21-year period, and all CAD events were validated using internationally standardized procedures [20, 24].

Additional support for a true leukocyte-CAD event association in PWH is provided by the increase in leukocyte count in CAD cases versus controls that can already be shown 8 years before the CAD event. This suggests the association of high leukocytes with CAD event risk is not attributable to short-term inflammatory/infectious illness immediately before the CAD event that might cause bursts of inflammation and thereby contribute to plaque rupture and CAD events. Our results stand in contrast mechanistically to the association in the general population of acute pneumonia or influenza with increased short-term CAD event risk [31]. Indirect support for the relevance of high leukocytes to CAD risk is afforded by data showing that adding leukocyte count to the Veterans Aging Cohort Study Index improved prediction of mortality [32].

In our Zurich subpopulation, high leukocytes had a larger CAD-odds ratio than high neutrophils. Leukocytes may provide a pathogenetic link between atherosclerosis and activation of pro-coagulatory mechanisms, and some general population literature [33, 34] points to a stronger neutrophil-CAD than leukocyte-CAD association [35]. However, the precise role of different leukocyte subtypes in predicting CAD events remains unresolved.

The leukocyte-CAD association was in part attenuated by smoking, a factor that is well-recorded to increase leukocytes, but less so by alcohol and black ethnicity, both of which may decrease leukocytes, or other factors with an established inflammatory link such as detectable HIV viremia or abdominal obesity.

Our study has limitations. Our population was 87% male, 94% white, and relatively young; therefore, results should only cautiously be extrapolated to other PWH. Leukocyte subtypes were available only in the Zurich participants, and insufficient information was available to analyze possible associations of leukocytes with chronic inflammatory conditions or corticosteroid therapy. Inflammatory markers such as high sensitivity C-reactive protein and interleukin-6 are not routinely measured in the SHCS. A potential link between inflammatory biomarkers and leukocytes would therefore be an important avenue for future investigation. Finally, we did not compare the leukocyte-CAD association in our PWH with a control population without HIV. However, the effect size of leukocytes on CAD risk that we report in PWH is very similar to effect sizes reported in the general population [14, 15].

In conclusion, we show how a high leukocyte count, most often in the normal range, may identify PWH at independently increased risk for CAD events. This increased risk persists after adjustment for traditional and HIV-related risk factors. Our findings expand on how inflammation (that may not yet be captured by current CAD risk assessment methods) may contribute to high leukocytes and CAD events in PWH.

## Supplementary Data

Supplementary materials are available at *Clinical Infectious Diseases* online. Consisting of data provided by the authors to benefit the reader, the posted materials are not copyedited and are the sole responsibility of the authors, so questions or comments should be addressed to the corresponding author.

## Supplementary Material

ciad033_Supplementary_Data

## Notes

***Author contributions*.** Study design: E. F. A., J. N. K., B. L., R. D. K., H. F. G., P. E. T. Data management, participant selection, case-control matching: B. L. Data acquisition: B. L., D. L. B., M. C. T., C. M., M. S., E. B., M. C., H. B., P. E. T. Data analysis: E. F. A., J. N. K., B. L., P. E. T. Drafting of the manuscript: E. F. A., B. L., P. E. T. Critical review and revision of the manuscript: all authors.

***Acknowledgments*.** The authors acknowledge the effort and commitment of investigators, study nurses, laboratory personnel, and participants.

***Swiss HIV Cohort Study (SHCS) members*.** Anagnostopoulos A, Battegay M, Bernasconi E, Boni J, Braun DL, Bucher HC, Calmy A, Cavassini M, Ciuffi A, Dollenmaier G, Egger M, Elzi L, Fehr J, Fellay J, Furrer H (chairman of the Clinical and Laboratory Committee), Fux CA, Gunthard HF (president of the SHCS), Haerry D (deputy of “Positive Council”), Hasse B, Hirsch HH, Hoffmann M, Hosli I, Huber M, Kahlert CR, Kaiser L, Keiser O, Klimkait T, Kouyos RD, Kovari H, Ledergerber B, Martinetti G, Martinez de Tejada B, Marzolini C, Metzner KJ, Muller N, Nicca D, Paioni P, Pantaleo G, Perreau M, Rauch A (chairman of the Scientific Board), Rudin C (chairman of the Mother & Child Substudy), Scherrer AU (head of Data Centre), Schmid P, Speck R, Stockle M, Tarr P, Trkola A, Vernazza P, Wandeler G, Weber R, Yerly S.

***Financial support*.** This work was supported by the SHCS [project 836], the Swiss National Science Foundation (grant number 201369), and the SHCS Research Foundation. SHCS data are gathered by the 5 Swiss university hospitals, 2 cantonal hospitals, 15 affiliated hospitals, and 36 private physicians (listed in http://www.shcs.ch/180-health-care-providers). B. L. reports support for this work from Kantonsspital Baselland (Data management and analyses).

## References

[1] Shah ASV , Stelzle D, Lee KK, et al Global burden of atherosclerotic cardiovascular disease in people living with HIV: systematic review and meta-analysis. Circulation 2018; 138:1100–12. 29967196 10.1161/CIRCULATIONAHA.117.033369 PMC6221183

[2] Feinstein MJ , Hsue PY, Benjamin LA, et al Characteristics, prevention, and management of cardiovascular disease in people living with HIV: a scientific statement from the American Heart Association. Circulation 2019; 140:e98–e124. 31154814 10.1161/CIR.0000000000000695 PMC7993364

[3] El-Sadr WM , Lundgren J, Neaton JD, et al CD4+ count-guided interruption of antiretroviral treatment. N Engl J Med 2006; 355:2283–96. 17135583 10.1056/NEJMoa062360

[4] Kuller LH , Tracy R, Belloso W, et al Inflammatory and coagulation biomarkers and mortality in patients with HIV infection. PLoS Med 2008; 5:e203. 18942885 10.1371/journal.pmed.0050203 PMC2570418

[5] Lang S , Mary-Krause M, Simon A, et al HIV replication and immune status are independent predictors of the risk of myocardial infarction in HIV-infected individuals. Clin Infect Dis 2012; 55:600–7. 22610928 10.1093/cid/cis489

[6] Silverberg MJ , Leyden WA, Xu L, et al Immunodeficiency and risk of myocardial infarction among HIV-positive individuals with access to care. J Acquir Immune Defic Syndr 2014; 65:160–6. 24442222 10.1097/QAI.0000000000000009

[7] Friis-Møller N , Reiss P, Sabin CA, et al Class of antiretroviral drugs and the risk of myocardial infarction. N Engl J Med 2007; 356:1723–35. 17460226 10.1056/NEJMoa062744

[8] Sabin CA , Worm SW, Weber R, et al Use of nucleoside reverse transcriptase inhibitors and risk of myocardial infarction in HIV-infected patients enrolled in the D:A:D study: a multi-cohort collaboration. Lancet 2008; 371:1417–26. 18387667 10.1016/S0140-6736(08)60423-7 PMC2688660

[9] Schoepf IC , Thorball CW, Ledergerber B, et al Coronary artery disease-associated and longevity-associated polygenic risk scores for prediction of coronary artery disease events in persons living with human immunodeficiency virus: the Swiss HIV Cohort Study. Clin Infect Dis 2021; 73:1597–604. 34091660 10.1093/cid/ciab521

[10] Baker JV , Neuhaus J, Duprez D, et al Inflammation predicts changes in high-density lipoprotein particles and apolipoprotein A1 following initiation of antiretroviral therapy. AIDS 2011; 25:2133–42. 21857489 10.1097/QAD.0b013e32834be088 PMC3320724

[11] Peters L , Neuhaus J, Duprez D, et al Biomarkers of inflammation, coagulation and microbial translocation in HIV/HCV co-infected patients in the SMART study. J Clin Virol 2014; 60:295–300. 24793968 10.1016/j.jcv.2014.03.017

[12] Friedman GD , Klatsky AL, Siegelaub AB. The leukocyte count as a predictor of myocardial infarction. N Engl J Med 1974; 290:1275–8. 4827627 10.1056/NEJM197406062902302

[13] Zalokar JB , Richard JL, Claude JR. Leukocyte count, smoking, and myocardial infarction. N Engl J Med 1981; 304:465–8. 7453772 10.1056/NEJM198102193040806

[14] Grimm RH Jr , Neaton JD, Ludwig W. Prognostic importance of the white blood cell count for coronary, cancer, and all-cause mortality. JAMA 1985; 254:1932–7. 4046122

[15] Danesh J , Collins R, Appleby P, Peto R. Association of fibrinogen, C-reactive protein, albumin, or leukocyte count with coronary heart disease: meta-analyses of prospective studies. JAMA 1998; 279:1477–82. 9600484 10.1001/jama.279.18.1477

[16] Freedman DS , Gates L, Flanders WD, et al Black/white differences in leukocyte subpopulations in men. Int J Epidemiol 1997; 26:757–64. 9279607 10.1093/ije/26.4.757

[17] Schwartz J , Weiss ST. Cigarette smoking and peripheral blood leukocyte differentials. Ann Epidemiol 1994; 4:236–42. 8055125 10.1016/1047-2797(94)90102-3

[18] Schwartz J , Weiss ST. Host and environmental factors influencing the peripheral blood leukocyte count. Am J Epidemiol 1991; 134:1402–9. 1776614 10.1093/oxfordjournals.aje.a116045

[19] Scherrer AU , Traytel A, Braun DL, et al Cohort profile update: the Swiss HIV Cohort Study (SHCS). Int J Epidemiol 2022; 51:33–4j. 34363666 10.1093/ije/dyab141

[20] World Health Organization . MONICA manual, part IV: event registration. Section 1: coronary event registration data component. 1999. Available at: https://www.thl.fi/publications/monica/manual/part4/iv-1.htm. Accessed 8 September 2020.

[21] Engel T , Raffenberg M, Schoepf IC, et al Telomere length, traditional risk factors, factors related to human immunodeficiency virus (HIV) and coronary artery disease events in Swiss persons living with HIV. Clin Infect Dis 2021; 73:e2070–e6. 32725240 10.1093/cid/ciaa1034

[22] Greenland S , Thomas DC. On the need for the rare disease assumption in case-control studies. Am J Epidemiol 1982; 116:547–53. 7124721 10.1093/oxfordjournals.aje.a113439

[23] Essebag V , Genest J Jr, Suissa S, Pilote L. The nested case-control study in cardiology. Am Heart J 2003; 146:581–90. 14564310 10.1016/S0002-8703(03)00512-X

[24] Ryom L , Lundgren JD, El-Sadr W, et al Cardiovascular disease and use of contemporary protease inhibitors: the D:A:D international prospective multicohort study. Lancet HIV 2018; 5:e291–300. 29731407 10.1016/S2352-3018(18)30043-2

[25] Tarr PE , Ledergerber B, Calmy A, et al Subclinical coronary artery disease in Swiss HIV-positive and HIV-negative persons. Eur Heart J 2018; 39:2147–54. 29590332 10.1093/eurheartj/ehy163

[26] Kovari H , Rauch A, Kouyos R, et al Hepatitis C infection and the risk of non-liver-related morbidity and mortality in HIV-infected persons in the Swiss HIV Cohort Study. Clin Infect Dis 2017; 64:490–7. 28172403 10.1093/cid/ciw809

[27] Combs JA , Norton EB, Saifudeen ZR, et al Human cytomegalovirus alters host cell mitochondrial function during acute infection. J Virol 2020; 94:e01183–19. 10.1128/JVI.01183-19 PMC6955246 31694945

[28] Petitti DB , Kipp H. The leukocyte count: associations with intensity of smoking and persistence of effect after quitting. Am J Epidemiol 1986; 123:89–95. 3940445 10.1093/oxfordjournals.aje.a114227

[29] Kannel WB , Anderson K, Wilson PW. White blood cell count and cardiovascular disease. Insights from the Framingham study. JAMA 1992; 267:1253–6. 1538564

[30] Dupont WD . Power calculations for matched case-control studies. Biometrics 1988; 44:1157–68. 3233252

[31] Musher DM , Rueda AM, Kaka AS, Mapara SM. The association between pneumococcal pneumonia and acute cardiac events. Clin Infect Dis 2007; 45:158–65. 17578773 10.1086/518849

[32] Tate JP , Sterne JAC, Justice AC; Veterans Aging Cohort Study (VACS) and the Antiretroviral Therapy Cohort Collaboration (ART-CC). Albumin, white blood cell count, and body mass index improve discrimination of mortality in HIV-positive individuals. AIDS 2019; 33:903–12. 30649058 10.1097/QAD.0000000000002140 PMC6749990

[33] Prentice RL , Szatrowski TP, Kato H, Mason MW. Leukocyte counts and cerebrovascular disease. J Chronic Dis 1982; 35:703–14. 7107804 10.1016/0021-9681(82)90094-7

[34] Welsh C , Welsh P, Mark PB, et al Association of total and differential leukocyte counts with cardiovascular disease and mortality in the UK biobank. Arterioscler Thromb Vasc Biol 2018; 38:1415–23. 29699973 10.1161/ATVBAHA.118.310945

[35] Libby P , Nahrendorf M, Swirski FK. Leukocytes link local and systemic inflammation in ischemic cardiovascular disease: an expanded “cardiovascular continuum”. J Am Coll Cardiol 2016; 67:1091–103. 26940931 10.1016/j.jacc.2015.12.048 PMC4779182

