# Supplementary material for: Leukocyte Count and Coronary Artery Disease Events in People With Human Immunodeficiency Virus: A Longitudinal Study
Source: Clin Infect Dis. 2023 Jan 23;76(11):1969–79. doi: 10.1093/cid/ciad033 (PMC10249993; doi:10.1093/cid/ciad033)
Supplement: ciad033_Supplementary_Data [file ciad033_supplementary_data.docx]

**SUPPLEMENTARY DATA**

Emma F. Avery et al

**Association of Leukocyte Count with Coronary Artery Disease Events in People Living with HIV: A Longitudinal Study**

**Supplementary Methods**

**Factors associated with leukocyte count**. We also considered injection drug use, educational level (mandatory school/apprenticeship/higher education/other) [1], season of leukocyte measurement (winter/spring/summer/fall) [2], body mass index (underweight/normal/overweight/obese) [3], and abdominal obesity (waist-hip-ratio ≥0.9 [men] and >0.85 [women]) [4].

**Supplementary Table 1: CAD Odds Ratios (95% Confidence Intervals) According to Leukocyte Quintiles and Clinical Variables, Univariable and Bivariable Analyses including potential interactions (likelihood ratio test)**

|  | **Univariable Analysis** | **Bivariable Analysis** | **Likelihood-Ratio Test for Interaction** |
| --- | --- | --- | --- |
| **Age per 10 years older** | 3.61 (1.98-6.61); p<0.01 | 3.97 (2.14-7.36); p<0.01 | p=0.28 |
| 1^st^ (lowest) leukocyte quintile | (reference) | (reference) |  |
| 2nd leukocyte quintile | 1.13 (0.80–1.59); p= 0.5 | 1.14 (0.80-1.61); p= 0.46 |  |
| 3rd leukocyte quintile | 1.44 (1.02–2.03); p= 0.04 | 1.46 (1.03-2.07); p= 0.03 |  |
| 4th leukocyte quintile | 1.70 (1.22–2.35); p<0.01 | 1.73 (1.24-2.42); p<0.01 |  |
| 5th (highest) leukocyte quintile | 2.27 (1.63–3.15); p<0.01 | 2.35 (1.69-3.26); p< 0.01 |  |
| **Ethnicity:** Black* | 0.55 (0.30–1.00); p= 0.05 | 0.67 (0.37–1.22); p= 0.19 | p=0.86 |
| **Ethnicity:** Hispanic* | 0.83 (0.30–2.33); p= 0.73 | 0.85 (0.30–2.39); p= 0.76 |  |
| **Ethnicity:** Asian* | 0.31 (0.09–1.08); p= 0.07 | 0.31 (0.09–1.07); p= 0.06 |  |
| 1^st^ (lowest) leukocyte quintile | (reference) | (reference) |  |
| 2nd leukocyte quintile | 1.13 (0.80–1.59); p= 0.5 | 1.11 (0.79–1.57); p= 0.55 |  |
| 3rd leukocyte quintile | 1.44 (1.02–2.03); p= 0.04 | 1.42 (1.01–2.01); p= 0.05 |  |
| 4th leukocyte quintile | 1.70 (1.22–2.35); p<0.01 | 1.66 (1.19-2.31); p<0.01 |  |
| 5th (highest) leukocyte quintile | 2.27 (1.63–3.15); p<0.01 | 2.21 (1.59–3.07); p<0.01 |  |
| **Risk Group:** IDU** | 1.38 (1.02–1.86); p= 0.04 | 1.28 (0.94–1.75); p= 0.11 | p=0.32 |
| **Risk Group:** HET** | 1.12 (0.86–1.44); p= 0.4 | 1.09 (0.84–1.41); p= 0.53 |  |
| **Risk Group:** other** | 0.72 (0.38–1.34); p= 0.3 | 0.66 (0.35–1.26); p= 0.21 |  |
| 1^st^ (lowest) leukocyte quintile | (reference) | (reference) |  |
| 2nd leukocyte quintile | 1.13 (0.80–1.59); p= 0.5 | 1.15 (0.82-1.63); p= 0.42 |  |
| 3rd leukocyte quintile | 1.44 (1.02–2.03); p= 0.04 | 1.47 (1.04-2.08); p= 0.03 |  |
| 4th leukocyte quintile | 1.70 (1.22–2.35); p<0.01 | 1.72 (1.23-2.39); p<0.01 |  |
| 5th (highest) leukocyte quintile | 2.27 (1.63–3.15); p<0.01 | 2.25 (1.62-3.13); p<0.01 |  |
| **Smoking:** Current smoking**** | 2.48 (1.87–3.28); p<0.01 | 2.14 (1.60–2.87); p<0.01 | p= 0.59 |
| **Smoking:** Past smoking**** | 1.48 (1.10–1.99); p= 0.01 | 1.44 (1.07–1.94); p= 0.02 |  |
| 1^st^ (lowest) leukocyte quintile | (reference) | (reference) |  |
| 2^nd^ leukocyte quintile | 1.13 (0.80–1.59); p= 0.50 | 1.11 (0.78–1.58); p= 0.55 |  |
| 3rd leukocyte quintile | 1.44 (1.02–2.03); p= 0.04 | 1.36 (0.96–1.92); p= 0.09 |  |
| 4th leukocyte quintile | 1.69 (1.22–2.35); p<0.01 | 1.48 (1.06–2.08); p= 0.02 |  |
| 5th (highest) leukocyte quintile | 2.27 (1.64–3.15); p<0.01 | 1.82 (1.30–2.56); p<0.01 |  |
| **Smoking:** No current smoking**** | 1.49 (1.11-2.01); p= 0.01 | 1.45 (1.07–1.96); p= 0.02 | p= 0.13 |
| **Smoking:** current ≤5 cigarettes/day**** | 1.59 (0.98–2.59); p= 0.06 | 1.40 (0.85–2.29); p= 0.18 |  |
| **Smoking:** current 6-20 cigarettes/day**** | 2.98 (2.19–4.05); p<0.01 | 2.61 (1.90–3.58); p<0.01 |  |
| **Smoking:** current >20 cigarettes/day**** | 2.05 (1.37–3.07); p<0.01 | 1.70 (1.12–2.57); p= 0.01 |  |
| **Smoking:** current unknown**** | 1.98 (0.73–5.40); p= 0.18 | 1.82 (0.67–4.99); p= 0.24 |  |
| 1st (lowest) leukocyte quintile | (reference) | (reference) |  |
| 2nd leukocyte quintile | 1.13 (0.80–1.59); p= 0.50 | 1.09 (0.77–1.56); p= 0.60 |  |
| 3rd leukocyte quintile | 1.44 (1.02–2.03); p= 0.04 | 1.39 (0.98–1.99); p= 0.06 |  |
| 4th leukocyte quintile | 1.69 (1.22–2.35); p<0.01 | 1.45 (1.04–2.04); p= 0.03 |  |
| 5th (highest) leukocyte quintile | 2.27 (1.64–3.15); p<0.01 | 1.85 (1.31–2.61); p<0.01 |  |
| **CMV seropositivity** | 1.35 (1.00–1.83); p= 0.05 | 1.43 (1.05-1.94); p= 0.02 | p= 0.98 |
| 1st (lowest) leukocyte quintile | (reference) | (reference) |  |
| 2nd leukocyte quintile | 1.13 (0.80–1.59); p= 0.5 | 1.12 (0.79-1.58); p= 0.52 |  |
| 3rd leukocyte quintile | 1.44 (1.02–2.03); p= 0.04 | 1.43 (1.01-2.03); p= 0.04 |  |
| 4th leukocyte quintile | 1.70 (1.22–2.35); p<0.01 | 1.70 (1.22-2.37); p<0.01 |  |
| 5th (highest) leukocyte quintile | 2.27 (1.63–3.15); p<0.01 | 2.30 (1.66-3.21); p< 0.01 |  |
| **HCV seropositivity** | 1.25 (0.97–1.61); p= 0.08 | 1.21 (0.93-1.57); p= 0.15 | p= 0.06 |
| 1st (lowest) leukocyte quintile | (reference) | (reference) |  |
| 2nd leukocyte quintile | 1.13 (0.80–1.59); p= 0.50 | 1.14 (0.81-1.61); p= 0.44 |  |
| 3rd leukocyte quintile | 1.44 (1.02–2.03); p= 0.04 | 1.47 (1.04-2.08); p= 0.03 |  |
| 4th leukocyte quintile | 1.69 (1.22–2.35); p<0.01 | 1.71 (1.23-2.39); p<0.01 |  |
| 5th (highest) leukocyte quintile | 2.27 (1.64–3.15); p<0.01 | 2.26 (1.63-3.13); p< 0.01 |  |
| **Dyslipidemia** | 1.58 (1.29–1.93); p<0.01 | 1.49 (1.21-1.83); p<0.01 | p= 0.17 |
| 1st (lowest) leukocyte quintile | (reference) | (reference) |  |
| 3rd leukocyte quintile | 1.13 (0.80–1.59); p= 0.50 | 1.41 (0.99-1.99); p= 0.06 |  |
| 4th leukocyte quintile | 1.44 (1.02–2.03); p= 0.04 | 1.61 (1.15-2.24); p<0.01 |  |
| 5th (highest) leukocyte quintile | 1.69 (1.22–2.35); p<0.01 | 2.15 (1.55-2.99); p< 0.01 |  |
| **Hypertension** | 1.40 (1.12–1.73); p<0.01 | 1.41 (1.13-1.76); p<0.01 | p= 0.21 |
| 1st (lowest) leukocyte quintile | (reference) | (reference) |  |
| 2nd leukocyte quintile | 1.13 (0.80–1.59); p= 0.5 | 1.11 (0.78-1.56); p= 0.57 |  |
| 3rd leukocyte quintile | 1.44 (1.02–2.03); p= 0.04 | 1.43 (1.01-2.02); p= 0.04 |  |
| 4th leukocyte quintile | 1.70 (1.22–2.35); p<0.01 | 1.67 (1.20-2.33); p<0.01 |  |
| 5th (highest) leukocyte quintile | 2.27 (1.63–3.15; p<0.01 | 2.28 (1.64-3.16); p< 0.01 |  |
| **BMI** Underweight | 1.65 (1.05–2.61); p= 0.03 | 1.49 (0.94-2.39); p= 0.09 | p= 0.13 |
| **BMI**: Overweight | 1.09 (0.87-1.36); p= 0.46 | 1.08 (0.86-1.36); p= 0.51 |  |
| **BMI**: Obese | 0.92 (0.63–1.35); p= 0.68 | 0.82 (0.56-1.22); p= 0.33 |  |
| 1st (lowest) leukocyte quintile | (reference) | (reference) |  |
| 2nd leukocyte quintile | 1.13 (0.80–1.59); p= 0.5 | 1.13 (0.80-1.60); p= 0.49 |  |
| 3rd leukocyte quintile | 1.44 (1.02–2.03); p= 0.04 | 1.45 (1.03-2.05); p= 0.04 |  |
| 4th leukocyte quintile | 1.70 (1.22–2.35); p<0.01 | 1.71 (1.23-2.39); p<0.01 |  |
| 5th (highest) leukocyte quintile | 2.27 (1.63–3.15); p<0.01 | 2.28 (1.64-3.17); p< 0.01 |  |
| **Abdominal Obesity (Waist-Hip Ratio ≥0.90 [men], >0.85 [women])** | 1.25 (1.08–1.45); p<0.01 | 1.20 (1.04-1.39); p= 0.02 | p= 0.29 |
| 1st (lowest) leukocyte quintile | (reference) | (reference) |  |
| 2nd leukocyte quintile | 1.13 (0.80–1.59); p= 0.5 | 1.16 (0.82-1.65); p= 0.4 |  |
| 3rd leukocyte quintile | 1.44 (1.02–2.03); p= 0.04 | 1.44 (1.02-2.04); p= 0.04 |  |
| 4th leukocyte quintile | 1.70 (1.22–2.35); p<0.01 | 1.71 (1.23-2.39); p<0.01 |  |
| 5th (highest) leukocyte quintile | 2.27 (1.63–3.15); p<0.01 | 2.25 (1.62-3.13); p< 0.01 |  |
| **Diabetes mellitus** | 2.19 (1.59–3.03); p<0.01 | 2.13 (1.53-2.95); p<0.01 | p= 0.55 |
| 1st (lowest) leukocyte quintile | (reference) | (reference) |  |
| 2nd leukocyte quintile | 1.13 (0.80–1.59); p= 0.5 | 1.11 (0.78-1.56); p=0.57 |  |
| 3rd leukocyte quintile | 1.44 (1.02–2.03); p= 0.04 | 1.43 (1.01-2.01); p=0.05 |  |
| 4th leukocyte quintile | 1.70 (1.22–2.35); p<0.01 | 1.68 (1.21-2.34); p<0.01 |  |
| 5th (highest) leukocyte quintile | 2.27 (1.63–3.15); p<0.01 | 2.20 (1.58-3.05); p<0.01 |  |
| **Family History of CAD** | 1.84 (1.38–2.45); p<0.01 | 1.82 (1.36-2.44); p<0.01 | p= 0.55 |
| 1st (lowest) leukocyte quintile | (reference) | (reference) |  |
| 2nd leukocyte quintile | 1.13 (0.80–1.59); p= 0.5 | 1.15 (0.81-1.62); p= 0.44 |  |
| 3rd leukocyte quintile | 1.44 (1.02–2.03); p= 0.04 | 1.43 (1.01-2.02); p= 0.04 |  |
| 4th leukocyte quintile | 1.70 (1.22–2.35); p<0.01 | 1.66 (1.19-2.32); p<0.01 |  |
| 5th (highest) leukocyte quintile | 2.27 (1.63–3.15); p<0.01 | 2.28 (1.64-3.17); p< 0.01 |  |
| **CD4 nadir <50cells/uL** | 1.20 (0.93–1.56); p= 0.16 | 1.22 (0.94-1.59); p= 0.14 | p= 0.95 |
| 1st (lowest) leukocyte quintile | (reference) | (reference) |  |
| 2nd leukocyte quintile | 1.13 (0.80–1.59); p= 0.5 | 1.12 (0.79-1.58); p= 0.52 |  |
| 3rd leukocyte quintile | 1.44 (1.02–2.03); p= 0.04 | 1.44 (1.02-2.03); p= 0.04 |  |
| 4th leukocyte quintile | 1.70 (1.22–2.35); p<0.01 | 1.69 (1.22-2.36); p<0.01 |  |
| 5th (highest) leukocyte quintile | 2.27 (1.63–3.15); p<0.01 | 2.27 (1.64-3.15); p< 0.01 |  |
| **Current HIV-RNA<50 copies/mL** | 0.92 (0.67–1.27); p= 0.61 | 0.90 (0.65-1.25); p= 0.53 | p= 0.33 |
| 1st (lowest) leukocyte quintile | (reference) | (reference) |  |
| 2nd leukocyte quintile | 1.13 (0.80–1.59); p= 0.5 | 1.12 (0.80-1.59); p= 0.51 |  |
| 3rd leukocyte quintile | 1.44 (1.02–2.03); p= 0.04 | 1.43 (1.02-2.03); p= 0.04 |  |
| 4th leukocyte quintile | 1.70 (1.22–2.35); p<0.01 | 1.70 (1.22-2.36); p<0.01 |  |
| 5th (highest) leukocyte quintile | 2.27 (1.63–3.15); p<0.01 | 2.27 (1.64-3.15); p< 0.01 |  |
| **On Abacavir** in the 6 months prior to CAD event | 1.73 (1.37–2.17); p<0.01 | 1.68 (1.34–2.12); p<0.01 | p= 0.55 |
| 1st (lowest) leukocyte quintile | (reference) | (reference) |  |
| 2nd leukocyte quintile | 1.13 (0.80–1.59); p= 0.5 | 1.07 (0.76–1.52); p= 0.69 |  |
| 3rd leukocyte quintile | 1.44 (1.02–2.03); p= 0.04 | 1.42 (1.00–2.01); p= 0.5 |  |
| 4th leukocyte quintile | 1.70 (1.22–2.35); p<0.01 | 1.64 (1.18–2.29); p<0.01 |  |
| 5th (highest) leukocyte quintile | 2.27 (1.63–3.15); p<0.01 | 2.17 (1.56–3.01); p<0.01 |  |
| **On Didanosine** in the 6 months prior | 1.81 (1.06-3.09); p=0.03 | 1.75 (1.02-3.01); p=0.04 | p= 0.52 |
| 1st (lowest) leukocyte quintile | (reference) | (reference) |  |
| 2nd leukocyte quintile | 1.13 (0.80–1.59); p= 0.5 | 1.12 (0.79–1.58); p= 0.53 |  |
| 3rd leukocyte quintile | 1.44 (1.02–2.03); p= 0.04 | 1.42 (1.01–2.01); p= 0.05 |  |
| 4th leukocyte quintile | 1.70 (1.22–2.35); p<0.01 | 1.67 (1.20–2.33); p<0.01 |  |
| 5th (highest) leukocyte quintile | 2.27 (1.63–3.15); p<0.01 | 2.25 (1.62–3.13); p<0.01 |  |
| **On Integrase-Inhibit** in the 6 months prior | 1.38 (1.03-1.85); p=0.03 | 1.35 (0.80-1.59); p=0.05 | p= 0.10 |
| 1st (lowest) leukocyte quintile | (reference) | (reference) |  |
| 2nd leukocyte quintile | 1.13 (0.80–1.59); p= 0.5 | 1.13 (0.80–1.59); p= 0.50 |  |
| 3rd leukocyte quintile | 1.44 (1.02–2.03); p= 0.04 | 1.45 (1.02–2.05); p= 0.04 |  |
| 4th leukocyte quintile | 1.70 (1.22–2.35); p<0.01 | 1.68 (1.21–2.34); p<0.01 |  |
| 5th (highest) leukocyte quintile | 2.27 (1.63–3.15); p<0.01 | 2.26 (1.63–3.13); p<0.01 |  |
| **Lopinavir/ritonavir,** exposure >1 year | 1.23 (0.98–1.56); p= 0.08 | 1.23 (0.97–1.56); p= 0.09 | p= 0.86 |
| 1st (lowest) leukocyte quintile | (reference) | (reference) |  |
| 2nd leukocyte quintile | 1.13 (0.80–1.59); p= 0.5 | 1.13 (0.80–1.59); p= 0.48 |  |
| 3rd leukocyte quintile | 1.44 (1.02–2.03); p= 0.04 | 1.44 (1.02–2.04); p= 0.04 |  |
| 4th leukocyte quintile | 1.70 (1.22–2.35); p<0.01 | 1.70 (1.22–2.37); p<0.01 |  |
| 5th (highest) leukocyte quintile | 2.27 (1.63–3.15); p<0.01 | 2.27 (1.64–3.15); p<0.01 |  |
| **Indinavir,** exposure >1 year | 1.12 (0.86–1.45); p= 0.41 | 1.12 (0.86–1.46); p= 0.39 | p= 1.00 |
| 1st (lowest) leukocyte quintile | (reference) | (reference) |  |
| 2nd leukocyte quintile | 1.13 (0.80–1.59); p= 0.5 | 1.12 (0.80–1.59); p= 0.51 |  |
| 3rd leukocyte quintile | 1.44 (1.02–2.03); p= 0.04 | 1.44 (1.02–2.03); p= 0.04 |  |
| 4th leukocyte quintile | 1.70 (1.22–2.35); p<0.01 | 1.70 (1.22–2.36); p<0.01 |  |
| 5th (highest) leukocyte quintile | 2.27 (1.63–3.15); p<0.01 | 2.27 (1.63–3.14); p<0.01 |  |
| **Darunavir,** exposure >1 year | 1.14 (0.85–1.54); p= 0.37 | 1.11 (0.82–1.49); p= 0.51 | p= 0.37 |
| 1st (lowest) leukocyte quintile | (reference) | (reference) |  |
| 2nd leukocyte quintile | 1.13 (0.80–1.59); p= 0.5 | 1.13 (0.80–1.59); p= 0.5 |  |
| 3rd leukocyte quintile | 1.44 (1.02–2.03); p= 0.04 | 1.44 (1.02–2.04); p= 0.04 |  |
| 4th leukocyte quintile | 1.70 (1.22–2.35; p<0.01 | 1.69 (1.21–2.35); p<0.01 |  |
| 5th (highest) leukocyte quintile | 2.27 (1.63–3.15); p<0.01 | 2.26 (1.63–3.14); p<0.01 |  |
| **Stavudine,** exposure >1 year | 1.55 (1.23–1.96); p<0.01 | 1.53 (1.21–1.95); p<0.01 | p= 0.18 |
| 1st (lowest) leukocyte quintile | (reference) | (reference) |  |
| 2nd leukocyte quintile | 1.13 (0.80–1.59); p= 0.5 | 1.09 (0.77–1.54); p= 0.63 |  |
| 3rd leukocyte quintile | 1.44 (1.02–2.03); p= 0.04 | 1.45 (1.03–2.05); p= 0.03 |  |
| 4th leukocyte quintile | 1.70 (1.22–2.35); p<0.01 | 1.68 (1.20–2.34); p<0.01 |  |
| 5th (highest) leukocyte quintile | 2.27 (1.63–3.15); p<0.01 | 2.21 (1.59–3.07); p<0.01 |  |
| **Last alcohol intake:** moderate/heavy***** | 0.81 (0.57–1.15); p= 0.24 | 0.80 (0.56–1.14); p= 0.21 | p= 0.35 |
| 1st (lowest) leukocyte quintile | (reference) | (reference) |  |
| 2nd leukocyte quintile | 1.13 (0.80–1.59); p= 0.5 | 0.99 (0.68–1.45); p= 0.97 |  |
| 3rd leukocyte quintile | 1.44 (1.02–2.03); p= 0.04 | 1.24 (0.85–1.80); p= 0.27 |  |
| 4th leukocyte quintile | 1.70 (1.22–2.35); p<0.01 | 1.47 (1.03–2.11); p=0.04 |  |
| 5th (highest) leukocyte quintile | 2.27 (1.63–3.15); p<0.01 | 1.98 (1.39–2.81); p<0.01 |  |
| **eGFR continuous******** | 1.15 (1.08-1.23); p<0.01 | 1.16 (1.08-1.24); p<0.01 | p= 0.52 |
| 1st (lowest) leukocyte quintile | (reference) | (reference) |  |
| 2nd leukocyte quintile | 1.02 (0.70–1.50); p= 0.91 | 0.98 (0.66–1.44); p= 0.90 |  |
| 3rd leukocyte quintile | 1.34 (0.92–1.97); p= 0.13 | 1.34 (0.91–1.96); p= 0.14 |  |
| 4th leukocyte quintile | 1.58 (1.09–2.28); p=0.02 | 1.55 (1.07–2.25); p=0.02 |  |
| 5th (highest) leukocyte quintile | 2.21 (1.55–3.17); p<0.01 | 2.19 (1.52–3.15); p<0.01 |  |

**Abbreviations.** BMI, body mass index; CAD, Coronary artery disease**;** CI, confidence interval; CKD, chronic kidney disease; CMV, cytomegalovirus; eGFR, estimated glomerular filtration rate; HET heterosexual; HCV, Hepatitis C Virus; IDU, intravenous drug use; OR, Odds Ratio

* White is the reference category

** Men who have sex with Men (MSM) are the reference category

*** mandatory school is the reference category

**** never smoked is the reference category

***** none/mild is the reference category

****** Reduced sample size: n= 445 cases vs n=1’101 controls

**Supplementary Table 2: Latest Leukocyte Count Quintiles, cells/uL, Median (Minimum-Maximum).**

|  | Cases | Controls | Total |
| --- | --- | --- | --- |
| 1st leukocyte quintile | 4125 (2150-4760) n=78 | 4180 (1480-4780) n=311 | 4150 (1480-4780) n=389 |
| 2nd leukocyte quintile | 5125 (4800-5600) n= 90 | 5240 (4800-5600) n=321 | 5200 (4800-5600) n=411 |
| 3rd leukocyte quintile | 6025 (5640-6490) n=100 | 6000 (5610-6490) n=280 | 6000 (5610-6490) n=380 |
| 4th leukocyte quintile | 7105 (6500-7810) n=124 | 7500 (6500-7800) n=296 | 7100 (6500-7810) n=420 |
| 5th leukocyte quintile | 9000 (7840- 20300) n=144 | 8900 (7830-60000) n=256 | 8960 (7830-60000) n=400 |
| Total | 6495 (2150-20300) n=536 | 5900 (1480-60000) n= 1464 | 6020 (1480-60000) n=2000 |

**Abbreviations.** n, number of participants

**Supplementary Table 3: Coronary Artery Disease (CAD) Odds Ratio (OR) According to Quintiles of Leukocyte Count and Individual Clinical Risk Factors. Univariable and Multivariable Analyses.**

NB: This table shows the same odds ratios, adjusted odds ratios and 95% confidence intervals (CI) that are illustrated in **Figure 4.**

|  | **Univariable analysis** | **Multivariable analysis** |
| --- | --- | --- |
| 1^st^ (lowest) leukocyte quintile* | (reference) | (reference) |
| 2nd leukocyte quintile* | 1.13 (0.80–1.59); p= 0.5 | 0.96 (0.66–1.38); p= 0.82 |
| 3rd leukocyte quintile* | 1.44 (1.02–2.03); p= 0.04 | 1.30 (0.90–1.90); p= 0.16 |
| 4th leukocyte quintile* | 1.70 (1.22–2.35); p<0.01 | 1.29 (0.90–1.85); p= 0.17 |
| 5th (highest) leukocyte quintile* | 2.27 (1.63–3.15); p<0.01 | 1.59 (1.09–2.30); p= 0.02 |
| **CD4 nadir** <50 cells/μL | 1.20 (0.93–1.56); p= 0.16 | 1.06 (0.79–1.41); p= 0.71 |
| On **Abacavir** in past 6 months | 1.73 (1.37–2.17); p<0.01 | 1.81 (1.41–2.33); p<0.01 |
| **On Didanosine** in past 6 months | 1.81 (1.06-3.09); p= 0.03 | 1.51 (0.84-2.70); p=0.17 |
| **Lopinavir/ritonavir, exposure** >1 year | 1.23 (0.98–1.56); p= 0.08 | 1.12 (0.86–1.45); p= 0.39 |
| **Stavudine, exposure** >1 year | 1.55 (1.23–1.96); p<0.01 | 1.34 (1.04–1.75); p= 0.03 |
| **On Integrase-Inhibitor** in the 6 past months | 1.38 (1.03-1.85); p= 0.03 | 1.16 (0.84-1.61); p=0.37 |
| **CMV Seropositivity** | 1.35 (1.00–1.83); p= 0.05 | 1.67 (1.19–2.34); p<0.01 |
| **Hepatitis C Seropositivity,** | 1.25 (0.97–1.61); p= 0.08 | 0.91 (0.61–1.35); p= 0.64 |
| **BMI** underweight | 1.65 (1.05–2.61); p= 0.03 | 1.42 (0.85–2.35); p=0.18 |
| **BMI** normal | (reference) | (reference) |
| **BMI** overweight | 1.09 (0.87-1.36); p= 0.46 | 1.08 (0.84–1.39); p=0.54 |
| **BMI** obese | 0.92 (0.63–1.35); p= 0.68 | 0.70 (0.46–1.08); p=0.11 |
| **Dyslipidemia** | 1.58 (1.29–1.93); p<0.01 | 1.44 (1.16–1.81); p<0.01 |
| **Hypertension** | 1.40 (1.12–1.73); p<0.01 | 1.54 (1.21–1.96); p<0.01 |
| **Diabetes** **mellitus** | 2.19 (1.59–3.03); p<0.01 | 2.15 (1.50–3.07); p<0.01 |
| **Family History of CAD** | 1.84 (1.38–2.45); p<0.01 | 1.60 (1.16–2.20); p<0.01 |
| **Age** at matching date (per 10 years) | 3.62 (1.98–6.61); p<0.01 | 4.37 (2.22–8.59); p<0.01 |
| **HIV acquisition group:** MSM | (reference) | (reference) |
| **HIV acquisition group:** Injection drug use | 1.38 (1.02–1.86); p= 0.04 | 1.21 (0.76–1.94); p= 0.42 |
| **HIV acquisition group:** heterosexual | 1.11 (0.86–1.44); p= 0.40 | 1.15 (0.85-1.55); p= 0.37 |
| **HIV acquisition group:** Other | 0.72 (0.38–1.34); p= 0.30 | 0.74 (0.38-1.45); p= 0.38 |
| **Ethnicity:** White | (reference) | (reference) |
| **Ethnicity:** Black | 0.55 (0.30–1.00); p= 0.05 | 0.63 (0.32-1.25); p= 0.19 |
| **Ethnicity:** Hispanic | 0.83 (0.30–2.33); p= 0.73 | 1.19 (0.40-3.60); p= 0.75 |
| **Ethnicity:** Asian | 0.31 (0.09–1.08); p= 0.07 | 0.31 (0.09-1.12); p= 0.07 |
| **Smoking Amount:** Never smoked | (reference) | (reference) |
| **Smoking Amount:** past smoker | 1.49 (1.11-2.01); p= 0.01 | 1.42 (1.03–1.96); p= 0.03 |
| **Smoking Amount:** Currently 1-5 cig/d | 1.59 (0.98–2.59); p= 0.06 | 1.42 (0.90-2.57); p= 0.12 |
| **Smoking Amount:** Currently 6-20 cig/d | 2.98 (2.19–4.05); p<0.01 | 2.77 (1.94–3.96); p<0.01 |
| **Smoking Amount:** Currently >20 cig/d | 2.05 (1.37–3.07); p<0.01 | 2.03 (1.29–3.19); p<0.01 |
| **Smoking Amount:** Current number not known | 1.98 (0.73–5.40); p= 0.18 | 1.78 (0.62–5.16); p= 0.28 |

**Additional variables, not included in final multivariable model:**

|  | **Univariable analysis** | **Multivariable analysis** |
| --- | --- | --- |
| **Waist/hip ratio** | 1.25 (1.08–1.45); p<0.01 | --*** |
| **Last alcohol intake: None/mild last** | (reference) | (reference) |
| **Last alcohol intake:** Moderate/heavy | 0.81 (0.57–1.15); p= 0.24 | --*** |
| **HIV RNA <50** copies/mL current | 0.92 (0.67–1.27); p= 0.61 | --*** |
| **Indinavir, exposure** >1 year | 1.12 (0.86–1.45); p= 0.41 | --*** |
| **Darunavir, exposure** >1 year**** | 1.15 (0.85–1.54); p= 0.37 | --*** |

**Abbreviations.** CAD, Coronary artery disease; Cig/d, cigarettes smoked per day**;** CMV, cytomegalovirus; MSM, men who have sex with men

* latest leukocyte count before CAD event (matching date)

** we included number of cigarettes smoked per day in the final model (rather than considering smoking as never/current/past) because of increased CAD event variability explained in univariable analysis (pseudo-R2 test, 3.88% vs. 3.42%) and in a bivariable model with latest leukocyte quintile (pseudo-R2 test, 4.99% vs. 4.48%)

*** were not included in the final model

**** Darunavir boosted by ritonavir or cobicistat

**Supplementary Table 4: Coronary Artery Disease (CAD) Odds Ratios (OR) and 95% Confidence Intervals (CI) According to Leukocyte Count at Different Intervals before CAD event (matching date), Univariable Analysis.**

|  | **Latest**  **Leukocyte Count** | **Year -1**  **Leukocyte Count** | **Year -2**  **Leukocyte Count** | **Year -3**  **Leukocyte Count** |
| --- | --- | --- | --- | --- |
| Definition in time, relative to CAD event | 180-1 day before CAD event | 180-540 days before CAD event | 540-910 days before CAD event | 910-1275 days before CAD event |
| Participants, n | 2000 | 1896 | 1749 | 1617 |
| Odds ratio for CAD event (95% confidence interval),  per 1000 leukocytes higher | 1.11 (1.05-1.16)  p<0.01 | 1.12 (1.06-1.17)  p<0.01 | 1.11 (1.06-1.17)  p<0.01 | 1.06 (1.01-1.12)  p=0.02 |
| Odds ratio for CAD event (95% confidence interval),  1st (lowest) leukocyte quintile | (reference) | (reference) | (reference) | (reference) |
| 2nd leukocyte quintile | 1.13 (0.80–1.59)  p= 0.50 | 1.02 (0.72–1.44)  p= 0.92 | 1.11 (0.78–1.57)  p= 0.56 | 1.06 (0.73–1.53);  p= 0.75 |
| 3nd leukocyte quintile | 1.44 (1.02–2.03)  p= 0.04 | 1.19 (0.84–1.67)  p= 0.34 | 1.04 (0.73–1.48)  p= 0.82 | 1.40 (0.97–2.00)  p= 0.07 |
| 4th leukocyte quintile | 1.69 (1.22–2.35)  p<0.01 | 1.35 (0.96–1.89)  p= 0.08 | 1.33 (0.94–1.88)  p= 0.10 | 1.22 (0.84–1.76)  p= 0.29 |
| 5th (highest) leukocyte quintile | 2.27 (1.64–3.15)  p<0.01 | 1.81 (1.30–2.53)  p<0.01 | 1.66 (1.18–2.33)  p<0.01 | 1.56 (1.09–2.22)  p= 0.02 |

|  | **Year -5**  **Leukocyte Count** | **Year -8**  **Leukocyte Count** | **Year -9**  **Leukocyte Count** | **Year -10**  **Leukocyte Count** |
| --- | --- | --- | --- | --- |
| Definition in time, relative to acute CAD event | 1645-2005 days before CAD event | 2740-3100 days before CAD event | 3105-3465 days before CAD event | 3470-3830 days before CAD event |
| Participants, n | 1231 | 657 | 499 | 369 |
| Odds ratio for CAD event (95% confidence interval),  per 1000 leukocytes higher | 1.07 (1.00-1.13)  p=0.04 | 1.16 (1.06-1.26)  p<0.01 | 1.08 (0.98-1.19)  p=0.13 | 0.97 (0.86-1.08)  p=0.55 |
| Odds ratio for CAD event (95% confidence interval),  1st (lowest) leukocyte quintile | (reference) | (reference) | (reference) | (reference) |
| 2nd leukocyte quintile | 1.19 (0.78–1.81)  p= 0.80 | 1.20 (0.66–2.19)  p= 0.55 | 1.06 (0.56–2.03)  p= 0.86 | 0.54 (0.26–1.16);  p= 0.11 |
| 3nd leukocyte quintile | 1.32 (0.88–2.00)  p= 0.18 | 1.16 (0.64–2.07)  p= 0.63 | 0.79 (0.39–1.59)  p= 0.51 | 0.81 (0.42–1.55)  p= 0.07 |
| 4th leukocyte quintile | 0.94 (0.61–1.44)  p=0.77 | 1.10 (0.61–1.98)  p= 0.75 | 1.19 (0.62–2.29)  p= 0.60 | 0.87 (0.44–1.74)  p= 0.70 |
| 5th (highest) leukocyte quintile | 1.74 (1.14–2.64)  p=0.01 | 2.18 (1.24–3.84)  p<0.01 | 1.12 (0.59–2.13)  p=0.72 | 0.66 (0.33–1.35)  p=0.26 |

**Supplementary Table 5: Latest Leukocyte and Neutrophil Count by different categories, Median (Interquartile Range).**

| **Leukocytes**  **Variable** | **Category** | **Latest Leukocyte Count, median (interquartile range), cells/uL**  **All participants** | **p-value** |
| --- | --- | --- | --- |
| Sex |  |  | P=0.70^a^ |
|  | Women (n=266) | 6140 (4900-7400) |  |
|  | Men (n= 1734) | 6015 (5010-7500) |  |
|  | Total (n=2000) | 6020 (5000-7460) |  |
| Age category at CAD/ matching date |  |  | P<0.01^a^ |
|  | 10-39 (n=102) | 5470 (4500-7000) |  |
|  | 40-49 (n=442) | 5985 (4800-7300) |  |
|  | 50-59 (n=769) | 6130 (5100-7600) |  |
|  | 60+ (n=687) | 6040 (5150-7300) |  |
|  | Total (n=2000) | 6020 (5000-7460) |  |
| Ethnicity |  |  | P<0.01^a^ |
|  | White (n=1876) | 6100 (5080-7500) |  |
|  | Black (n=78) | 4925 (4000-5800) |  |
|  | Hispanic (n=19) | 6100 (4700-7900) |  |
|  | Asian (n=27) | 7000 (5310-7900) |  |
|  | Total (n=2000) | 6020 (5000-7460) |  |
| Alcohol intake |  |  | P=0.28^a^ |
|  | None/mild (n=1492) | 6095 (5050-7500) |  |
|  | Moderate/heavy (=203) | 6300 (5100-7700) |  |
|  | Total (n=1695) | 6100 (5080-7500) |  |
| Smoking status |  |  | P<0.01^a^ |
|  | Never (n=554) | 5600 (4740-65600) |  |
|  | Current (n=847) | 6800 (5460-8290) |  |
|  | Past (n=599) | 5800 (4900-7000) |  |
|  | Total (n=2000) | 6020 (5000-7460) |  |
| Smoking amount |  |  | P<0.01^a^ |
|  | Never smoked (n=753) | 5630 (4780-6620) |  |
|  | Not currently (n=400) | 5800 (4900-6980) |  |
|  | ≤5 cpd (n=113) | 6400 (5300-7900) |  |
|  | 6-20 cpd (n=527) | 6800 (5400-8200) |  |
|  | >20 cpd (n=183) | 7100 (6000-8800) |  |
|  | Unknown (n=24) | 5610 (5050-7075) |  |
|  | Total (n=2000) | 6020 (5000-7460) |  |
| BMI |  |  | P<0.01^a^ |
|  | Underweight (n=90) | 6575 (5200-8200) |  |
|  | Normal (n=1095) | 5900 (4900-7380) |  |
|  | Overweight (=637) | 6000 (5100-7700) |  |
|  | Obese (n=177) | 6500 (5400-7700) |  |
|  | Total (n=1999) | 6020 (5000-7460) |  |
| Diabetes |  |  | P=0.33^a^ |
|  | Without (n=1801) | 6000 (5000-7400) |  |
|  | With (n=199) | 6020 (5000-7460) |  |
|  | Total (n=2000) | 6020 (5000-7460) |  |
| Hypertension |  |  | P=0.37^a^ |
|  | Without (n=1387) | 6000 (4980-7460) |  |
|  | With (n=613) | 6020 (5100-7460) |  |
|  | Total (n=2000) | 6020 (5000-7460) |  |
| Dyslipidemia |  |  | P<0.01^a^ |
|  | Without (n=974) | 5800 (4900-7100) |  |
|  | With (n=1026) | 6300 (5200-7700) |  |
|  | Total (n=2000) | 6020 (5000-7460) |  |
| Season |  |  | P=0.86^a^ |
|  | Spring (n=477) | 5900 (5000-7300) |  |
|  | Summer (n=507) | 6020 (5000-7500) |  |
|  | Fall (n=550) | 6100 (5000-7500) |  |
|  | Winter (n=466) | 6100 (5100-7500) |  |
|  | Total (n=2000) | 6020 (5000-7460) |  |
| Education level |  |  | P<0.01^a^ |
|  | Mandatory school (n=331) | 6200 (4950-7700) |  |
|  | Apprenticeship (n=947) | 6200 (5100-7600) |  |
|  | Higher education (n=619) | 5800 (4970-7000) |  |
|  | Other/missing (n=103) | 5800 (4900-7200) |  |
|  | Total (n=2000) | 6020 (5000-7460) |  |
| Opportunistic diseases in 1 year prior to CAD event* |  |  | P=0.64^b^ |
|  | Without (n=1962) | 6025 (5010-7440) |  |
|  | With (n=38) | 5985 /4400-7900) |  |
|  | Total (n=2000) | 6020 (5000-7460) |  |
| Serious non-opportunistic infections in 1 year prior to CAD event ** |  |  | P=0.59^b^ |
|  | Without (n=574) | 6200 (5220-7640) |  |
|  | With (n=36) | 5900 (5020-7550) |  |
|  | Total (n=610) | 6020 (5000-7460) |  |
|  |  |  |  |
| **Neutrophils***** | **Category** | **Latest Neutrophil Count, median (interquartile range) cells/mm3** | P<0.01^b^ |
|  | Cases (n=132) | 3835 (2800-4925) |  |
|  | Controls (n=385) | 3220 (2470-4230) |  |
|  | Total (n=517) | 3370 (2560-4380) |  |

**Abbreviations:** CAD, Coronary artery disease; CI, confidence interval; CPD, cigarettes smoked per day; OR, Odds Ratio; SNOI, serious non-opportunistic infections

* defined as CDC Stage B or C events

** captured in SHCS database since September 2017

*** n=517 University of Zurich participants only

^a^ Kruskal-Wallis Test

^b^ Wilcoxon rank-sum Test

**Supplementary Table 6: Sensitivity Analysis: CAD Odds Ratio (95% Confidence Interval) According to Quintiles of Leukocyte Count**

**Panel A: Adjustment for Framingham Risk Score per percentage point increase instead of Clinical Risk Factors, Uni- and Bivariable Analysis**

**Panel B: Adjustment for Framingham Risk Score Category (>10% vs. <10%) instead of Clinical Risk Factors, Uni- and Bivariable Analysis**

**A)**

|  | **Univariable** | **Bivariable** |
| --- | --- | --- |
| 1^st^ (lowest) leukocyte quintile | (reference) | (reference) |
| 2^nd^ leukocyte quintile | 1.13 (0.80–1.59); p= 0.5 | 0.99 (0.69-1.41); p=0.95 |
| 3^rd^ leukocyte quintile | 1.44 (1.02–2.03); p= 0.04 | 1.24 (0.86-1.78); p=0.24 |
| 4^th^ leukocyte quintile | 1.70 (1.22–2.35); p<0.01 | 1.27 (0.89-1.81); p=0.18 |
| 5th (highest) leukocyte quintile | 2.27 (1.63–3.15); p<0.01 | 1.64 (1.16-2.32); p<0.01 |
| **Framingham Risk Score (per percentage point increase)** | 1.09 (1.08-1.11); p<0.01 | 1.09 (1.07-1.11); p<0.01 |

**B)**

|  | **Univariable** | **Bivariable** |
| --- | --- | --- |
| 1^st^ (lowest) leukocyte quintile | (reference) | (reference) |
| 2^nd^ leukocyte quintile | 1.13 (0.80–1.59); p= 0.5 | 1.04 (0.73-1.48); p=0.82 |
| 3^rd^ leukocyte quintile | 1.44 (1.02–2.03); p= 0.04 | 1.36 (0.95-1.93); p=0.09 |
| 4^th^ leukocyte quintile | 1.70 (1.22–2.35); p<0.01 | 1.37 (0.97-1.93); p=0.07 |
| 5th (highest) leukocyte quintile | 2.27 (1.63–3.15); p<0.01 | 1.82 (1.30-2.56); p<0.01 |
| **Framingham Risk Score (>10% vs. <10%)** | 2.90 (2.31-3.63); p<0.01 | 2.68 (2.12-3.37); p<0.01 |

**Abbreviations**: CAD, coronary artery disease; CI, confidence interval; OR, Odds Ratio

**Supplementary Table 7: Sensitivity Analysis: CAD Odds Ratio (95% Confidence Interval) Restricted to Participants with Suppressed HIV RNA (1559/2000 study participants)**

|  | **Univariable analysis** | **Multivariable analysis** |
| --- | --- | --- |
| 1^st^ (lowest) leukocyte quintile* | (reference) | (reference) |
| 2nd leukocyte quintile* | 1.01 (0.68–1.51); p= 0.94 | 0.87 (0.57–1.34); p= 0.54 |
| 3rd leukocyte quintile* | 1.49 (1.00–2.00); p= 0.05 | 1.41 (0.92–2.17); p= 0.11 |
| 4th leukocyte quintile* | 1.61 (1.10–2.34); p=0.01 | 1.26 (0.83–1.91); p= 0.28 |
| 5th (highest) leukocyte quintile* | 2.17 (1.50–3.15); p<0.01 | 1.63 (1.06–2.50); p= 0.03 |
| **CD4 nadir <50 cells/μL** | 1.11 (0.83–1.48); p= 0.49 | 1.00 (0.72–1.39); p= 0.98 |
| On **Abacavir** in the 6 months prior | 1.66 (1.30–2.12); p<0.01 | 1.75 (1.33–2.30); p<0.01 |
| **On Didanosine** in the 6 months prior | 1.92 (0.98-3.78); p= 0.06 | 1.64 (0.78-3.42); p=0.19 |
| **Lopinavir/ritonavir, exposure** >1 year | 1.11 (0.86–1.43); p= 0.44 | 1.00 (0.75–1.33); p= 0.99 |
| **Stavudine, exposure** >1 year | 1.42 (1.09–1.984; p=0.01 | 1.30 (0.97–1.75); p= 0.08 |
| **On Integrase-Inhibit** in the 6 months prior | 1.44 (1.06-1.84); p= 0.01 | 1.24 (0.89-1.74); p=0.21 |
| **CMV Seropositivity** | 1.38 (0.98–1.93); p= 0.06 | 1.66 (1.14–2.44); p<0.01 |
| **Hepatitis C Seropositivity,** | 1.18 (0.89–1.57); p= 0.26 | 0.95 (0.60–1.52); p= 0.84 |
| **BMI** underweight | 1.56 (0.92–2.66); p= 0.10 | 1.32 (0.72–2.42); p= 0.36 |
| **BMI** normal | (reference) | (reference) |
| **BMI** overweight | 1.09 (0.85-1.41); p= 0.48 | 1.07 (0.81–1.42); p= 0.64 |
| **BMI** obese | 0.82 (0.54–1.26); p= 0.37 | 0.63 (0.39–1.02); p= 0.06 |
| **Dyslipidemia** | 1.60 (1.28–2.00); p<0.01 | 1.45 (1.13–1.87); p<0.01 |
| **Hypertension** | 1.25 (0.99–1.58); p=0.07 | 1.41 (1.08–1.84); p=0.01 |
| **Diabetes** **mellitus** | 2.07 (1.45–2.95); p<0.01 | 2.13 (1.44–3.17); p<0.01 |
| **Family History of CAD** | 1.88 (1.36–2.61); p<0.01 | 1.75 (1.22–2.52); p<0.01 |
| **Age** at matching date (per 10 years) | 3.40 (1.73–6.68); p<0.01 | 4.74 (2.20–10.22); p<0.01 |
| **HIV acquisition group:** MSM | (reference) | (reference) |
| **HIV acquisition group:** Injection drug use | 1.19 (0.85–1.68); p= 0.30 | 1.03 (0.60–1.79); p= 0.36 |
| **HIV acquisition group:** heterosexual | 1.04 (0.78–1.39); p= 0.77 | 1.10 (0.78-1.53); p= 0.59 |
| **HIV acquisition group:** Other | 0.65 (0.27–1.39); p= 0.27 | 0.75 (0.33-1.70); p= 0.49 |
| **Ethnicity:** White | (reference) | (reference) |
| **Ethnicity:** Black | 0.58 (0.30–1.11); p= 0.10 | 0.70 (0.33-1.50); p= 0.36 |
| **Ethnicity:** Hispanic | 0.97 (0.32–2.92); p= 0.96 | 1.10 (0.33-3.72); p= 0.88 |
| **Ethnicity:** Asian | 0.23 (0.05–1.00); p= 0.05 | 0.20 (0.04-0.93); p= 0.04 |
| **Smoking Amount:** Never smoked | (reference) | (reference) |
| **Smoking Amount:** not currently | 1.44 (1.04-1.99); p= 0.03 | 1.34 (0.94–1.91); p= 0.11 |
| **Smoking Amount:** Currently ≤5 cig/d | 1.43 (0.82–2.46); p= 0.20 | 1.33 (0.73-2.40); p= 0.35 |
| **Smoking Amount:** Currently 6-20 cig/d | 2.74 (1.96–3.83); p<0.01 | 2.52 (1.70–3.74); p<0.01 |
| **Smoking Amount:** Currently >20 cig/d | 1.64 (1.55–3.62); p<0.01 | 1.70 (0.98–2.93); p=0.06 |
| **Smoking Amount:** Current number not known | 1.98 (0.73–5.40); p= 0.18 | 0.65 (0.12–3.44); p= 0.62 |

**Abbreviations.** CAD, Coronary artery disease; Cig/d, cigarettes smoked per day**;** CMV, cytomegalovirus; MSM, men who have sex with men

* latest leukocyte count before CAD event (matching date)

**Supplementary Table 8: CAD Odds Ratio (95% Confidence Interval) According to Neutrophil and Leukocyte Quintiles in 517 University Zurich Participants, Univariable Analysis**

|  | **Univariable CAD odds ratio (95% CI)** |
| --- | --- |
| 1^st^ (lowest) **neutrophil** quintile | (reference) |
| 2^nd^ neutrophil quintile | 0.93 (0.45-1.90); p=0.84 |
| 3^rd^ neutrophil quintile | 0.80 (0.38-1.68); p=0.56 |
| 4^th^ neutrophil quintile | 2.17 (1.12-4.18); p=0.02 |
| 5^th^ (highest) neutrophil quintile | 2.19 (1.13-4.26); p=0.02 |
|  |  |
| 1^st^ (lowest) **leukocyte** quintile | (reference) |
| 2^nd^ leukocyte quintile | 2.39 (1.16-4.93); p=0.02 |
| 3^rd^ leukocyte quintile | 1.93 (0.90-4.17); p=0.09 |
| 4^th^ leukocyte quintile | 3.04 (1.41-6.56); p<0.01 |
| 5^th^ (highest) leukocyte quintile | 4.78 (2.31-9.87); p<0.01 |

**Abbreviations**: CI, confidence interval; OR, Odds Ratio

**Supplementary Table 9: Sensitivity Analysis: CAD Odds Ratio (95% Confidence Interval) According to Quintiles of Leukocyte Count, in 1546 Participants, Including Latest Estimated Glomerular Filtration Rate**

**Panel A: Uni- and Bivariable Analysis**

**Panel B: Final Multivariable Model with latest eGFR**

Note: Reduced number of study participants (n=1546) and reduced study period (1.1.2002 to 31.10.2021), because kidney function included in SHCS database only since 1.1.2002

**A)**

|  | **Univariable Analysis** | **Bivariable Analysis** |
| --- | --- | --- |
| **Latest Estimated glomerular filtration rate prior to CAD event (per 1 mL/min/1.73m^2^ lower)** | 1.15 (1.08-1.23); p<0.01 | 1.16 (1.09-1.24); p<0.01 |
| 1^st^ (lowest) leukocyte quintile | (reference) | (reference) |
| 2nd leukocyte quintile | 1.02 (0.70-1.50); p=0.91 | 0.98 (0.66-1.44); p=0.90 |
| 3rd leukocyte quintile | 1.34 (0.92-1.97); p=0.13 | 1.34 (0.91-1.96); p=0.14 |
| 4th leukocyte quintile | 1.58 (1.09-2.28); p=0.02 | 1.55 (1.07-2.25); p=0.02 |
| 5th (highest) leukocyte quintile | 2.21 (1.55-3.17); p<0.01 | 2.19 (1.52-3.15); p<0.01 |

**B)**

|  | **Multivariable analysis** |
| --- | --- |
| **1^st^ (lowest) leukocyte quintile** | (reference) |
| **2nd leukocyte quintile** | 0.84 (0.55-1.27); p=0.40 |
| **3rd leukocyte quintile** | 1.19 (0.78-1.81); p=0.42 |
| **4th leukocyte quintile** | 1.19 (0.79-1.79); p=0.40 |
| **5th (highest) leukocyte quintile** | 1.52 (1.00-2.31); p=0.05 |
| Latest **estimated glomerular filtration rate** prior to CAD event (per 1 mL/min/1.73m^2^ lower) | 1.13 (1.05-1.21); p<0.01 |
| **CD4 nadir** **<50 cells/μL** | 1.14 (0.82-1.59); p=0.42 |
| **HIV RNA <50** copies/mL current | 0.96 (0.63-1.46); p=0.85 |
| **Abacavir** in the 6 months prior | 1.64 (1.23-2.18); p<0.01 |
| **Didanosine** in the 6 months prior | 2.92 (1.33-6.39); p<0.01 |
| **Lopinavir/ritonavir, exposure** >1 year | 1.14 (0.85-1.51); p=0.39 |
| **Indinavir, exposure** >1 year | 1.01 (0.73-1.40); p=0.94 |
| **Darunavir, exposure** >1 year | 0.90 (0.62-1.29); p=0.56 |
| **Stavudine, exposure** >1 year | 1.43 (1.06-1.92); p=0.02 |
| **Integrase Inhibitor** in the 6 months prior | 1.08 (0.75-1.54); p=0.69 |
| **CMV Seropositivity** | 1.60 (1.08-2.37); p=0.02 |
| **Hepatitis C Seropositivity** | 0.99 (0.63-1.569; p=0.97 |
| **Dyslipidemia** | 1.43 (1.11-1.84); p<0.01 |
| **Hypertension** | 1.43 (1.09-1.88); p=0.01 |
| **Diabetes** **mellitus** | 1.73 (1.17-2.56); p<0.01 |
| **Family History of CAD** | 1.69 (1.17-2.43); p<0.01 |
| **Age** at matching date (per 10 years) | 4.62 (2.10-10.18); p<0.01 |
| **HIV acquisition group:** MSM | (reference) |
| **HIV acquisition group:** Injection drug use | 1.13 (0.65-1.97); p=0.66 |
| **HIV acquisition group:** heterosexual | 1.16 (0.82-1.62); p=0.40 |
| **HIV acquisition group:** Other | 0.90 (0.43-1.91); p=0.80 |
| **Ethnicity:** White | (reference) |
| **Ethnicity:** Black | 1.03 (0.50-2.15); p=0.93 |
| **Ethnicity:** Hispanic | 1.02 (0.29-3.67); p=0.97 |
| **Ethnicity:** Asian | 0.33 (0.09-1.23); p=0.10 |
| **Smoking:** Never smoked | (reference) |
| **Smoking:** not currently | 1.54 (1.10-2.219; p=0.02 |
| **Smoking:** Currently ≤5 cig/d | 1.73 (0.97-3.10); p=0.06 |
| **Smoking:** Currently 6-20 cig/d | 2.96 (1.98-4.42); p<0.01 |
| **Smoking:** Currently >20 cig/d | 2.21 (1.31-3.71); p<0.01 |
| **Smoking:** Current number of cig/d not known | 3.34 (0.42-26.80); p=0.26 |

**Abbreviations**: CIG; cigarettes; CMV, cytomegalovirus; D, day; MSM, men who have sex with men;

**Supplementary Figure 1: Descriptive Longitudinal Trends for Leukocyte Count (Panel A), Leukocyte subsets (Panels B-D), HIV-RNA (Panel E), Leukocytes Stratified by Smoking (Panel F) and for Neutrophil Count (Panel G) in Cases and Controls.**

**A: Total Leukocyte Count**

**
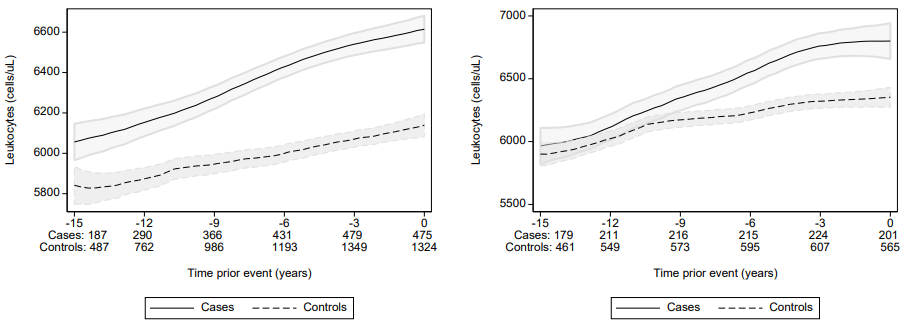
**

**B: Lymphocyte Count**

**
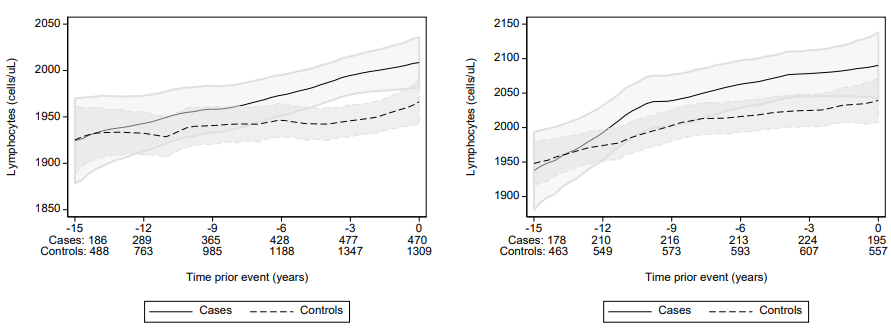
**

**C: CD4 count

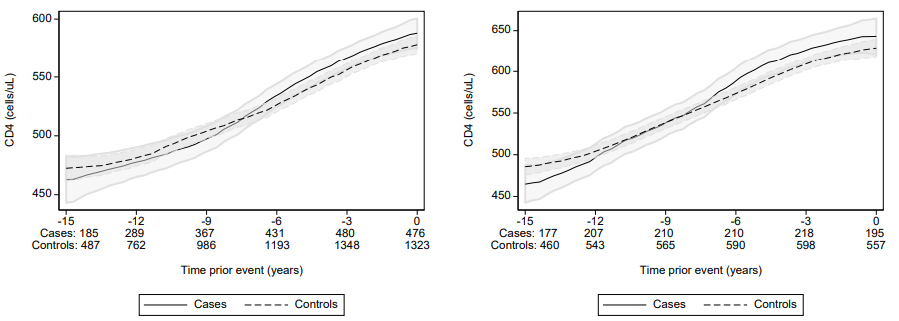
**

**D: CD8 count**


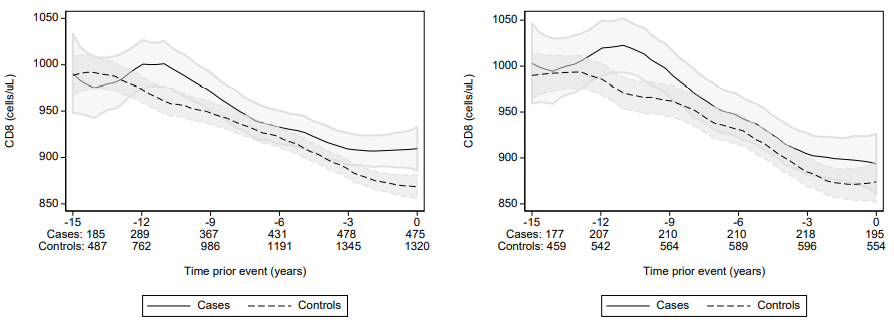


**E: HIV-RNA**


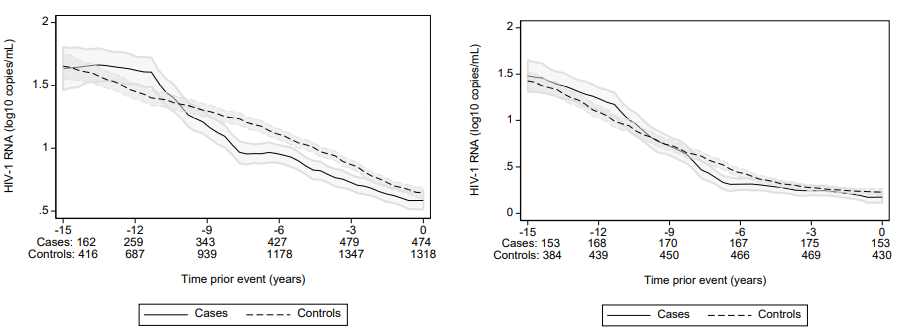


**F: Leukocyte Count Stratified by Smoking Status**


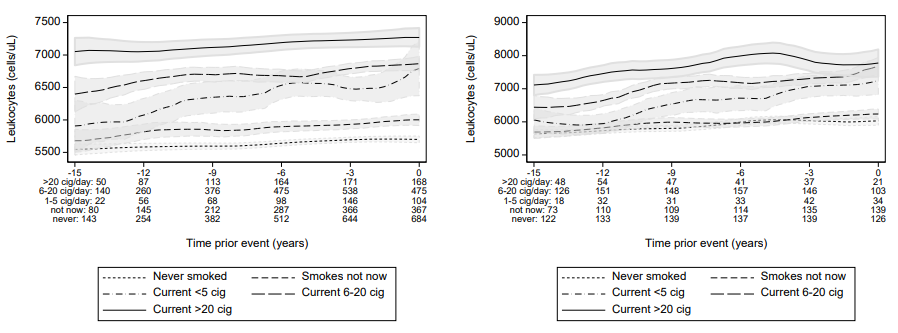


**G: Neutrophil Count (517 University of Zurich participants)**

**
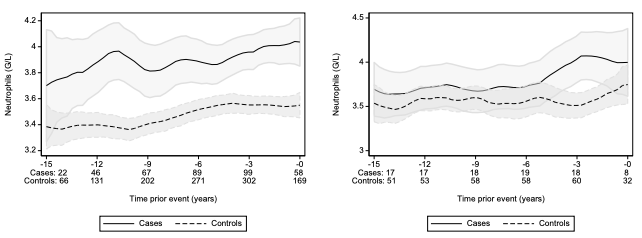
**

In these graphs we depict the descriptive (observed) longitudinal trajectories of total leukocytes and different leukocyte subtypes over time for controls vs. cases. The lines show the median leukocyte count and the shaded areas denote the 95% confidence intervals. We considered only parameters that were from the regular (per protocol) 6-monthly follow-up SHCS visits up until 1 day prior to the CAD event (cases) and matching date (controls). The graphs on the left portray an open cohort design (all participants are included, irrespective of observation duration), whereas the graphs on the right portray a closed cohort (only participants with >15 years observation time are included). [5]

**Abbreviations**: CAD, Coronary artery disease; CIG, cigarettes; ROC AUC, area under the receiver operating characteristic curve. SHCS, Swiss HIV Cohort Study

References

1. Pollitt RA, Kaufman JS, Rose KM, Diez-Roux AV, Zeng D, Heiss G. Early-life and adult socioeconomic status and inflammatory risk markers in adulthood. Eur J Epidemiol **2007**; 22(1): 55-66.

2. Wyse C, O'Malley G, Coogan AN, McConkey S, Smith DJ. Seasonal and daytime variation in multiple immune parameters in humans: Evidence from 329,261 participants of the UK Biobank cohort. iScience **2021**; 24(4): 102255.

3. Zhang SS, Yang XJ, Ma QH, et al. Leukocyte related parameters in older adults with metabolically healthy and unhealthy overweight or obesity. Sci Rep **2021**; 11(1): 4652.

4. Barcelo C, Guidi M, Thorball CW, et al. Impact of Genetic and Nongenetic Factors on Body Mass Index and Waist-Hip Ratio Change in HIV-Infected Individuals Initiating Antiretroviral Therapy. Open Forum Infect Dis **2020**; 7(1): ofz464.

5. Ledergerber B, Cavassini M, Battegay M, et al. Trends over time of virological and immunological characteristics in the Swiss HIV Cohort Study. HIV Med **2011**; 12(5): 279-88.
